# Inventory of the carabid beetle fauna of the Gaoligong Mountains, western Yunnan Province, China: species of the tribe Zabrini (Coleoptera, Carabidae)

**DOI:** 10.3897/zookeys.407.7353

**Published:** 2014-05-08

**Authors:** David H. Kavanaugh, Fritz Hieke, Hongbin Liang, Dazhi Dong

**Affiliations:** 1 Department of Entomology, California Academy of Sciences, 55 Music Concourse Drive, Golden Gate Park, San Francisco, CA 94118, U.S.A.; 2 Museum für Naturkunde, Invalidenstrasse 43, D-10115 Berlin, Germany; 3 Key Laboratory of Zoological Systematics, Institute of Zoology, Chinese Academy of Sciences, Beijing 100101, China; 4 Kunming Institute of Zoology, Chinese Academy of Sciences, Kunming 65022 Yunnan, China

**Keywords:** Coleoptera, Carabidae, Zabrini, *Amara*, China, Yunnan, Gaoligong Shan, Himalaya, Qinghai-Xizang (Tibetan) Plateau, distribution, biodiversity hotspot

## Abstract

A ten-year multidisciplinary, multi-national and multi-institutional biodiversity inventory project in the Gaoligong Shan region of western Yunnan Province, China generated more than 35,000 specimens of the beetle (Coleoptera) family Carabidae. In this report, first of a planned series, we focus on diversity in tribe Zabrini. Our study of just over 1300 specimens of zabrine carabids from the project, all in genus *Amara* Bonelli, found a total of 13 species, all previously described, to occur in the study area, with none of them strictly endemic. We present a key for identification of adults of these species, as well as nomenclatural data, diagnoses, illustrations of dorsal habitus and male genitalia, and information about geographical, altitudinal and habitat distributions within the study area and overall geographical distribution for each species. Distributions of the species within the study area are compared, and broader geographical range patterns are characterized. We also discuss a possible role of the Gaoligong Shan region as one source area for the present-day fauna of the Himalaya and southern edge of the Qinghai-Xizang (Tibetan) Plateau.

## Introduction

**Figure 1.**
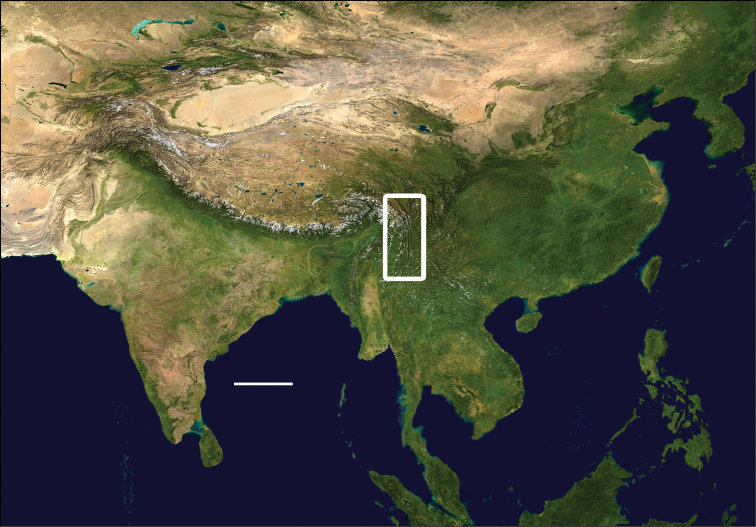
Map of Asia with study region outlined; scale line = 500 km. Modified from Wikimedia Commons, World Atlas of the World, at URL: http://upload.wikimedia.org/wikipedia/commons/8/8f/Whole_world_-_land_and_oceans_12000.jpg

The Gaoligong Shan (Gaoligong Mountains) of extreme western Yunnan province, China, form the westernmost range of the Hengduan Mountains system of southeastern Xizang Autonomous Region (Tibet), northern and western Yunnan, and western Sichuan (Fig. 1). They extend north to south for more than 600 km, and, in the central part of the range, their crest forms the border between China and Myanmar. They also separate and form parts of the watersheds of two of Southeast Asia’s major rivers, the Irrawaddy and the Salween (known in China as the Nujiang). Elevations within the region range from a low of about 650 m in the south to more than 5000 m in the north. Chaplin (2006) reviewed the physical geography of the region. Because of its geographic isolation and rugged topography, much of this area has remained less disturbed than most other parts of China; and previous biological exploration of the area over the past 150 years has revealed exceptionally high species richness, based almost exclusively on records for vertebrates (e.g., Stattersfield et al. 1998) and vascular plants (Li et al 2000). Because of these traits, two large nature reserves have been established in the area, and the region has been included in the Three Parallel Rivers of Yunnan World Heritage Site (UNESCO 2003).

In late 1997, the California Academy of Sciences was invited to participate in a joint project with the Kunming Institutes of Botany and Zoology of the Chinese Academy of Sciences to conduct a biodiversity inventory of the Gaoligong Mountains. Scientists from several additional institutions, including the Institute of Zoology, Beijing, and Royal Botanical Garden (Edinburgh) joined in the collaboration. Principal target groups for the inventory included bryophytes and vascular plants, all vertebrate groups, and arachnids, myriapods, and insects, especially the Neuropteroidea, Mecoptera, and Coleoptera (the Carabidae in particular). Multidisciplinary and multi-institutional teams carried out biotic sampling through more than 25 separate expeditions during the period 1998 to 2007. More than 100 reports on the project have been published to date, including partial results for bryophytes (e.g., Long 2006, Shevock 2005), plants (e.g., Fritsch et al. 2008, Zhou et al. 2006), birds (Dumbacher et al. 2011), amphibians (e.g., Liu et al. 2000), fishes (e.g., Chen et al. 2005), spiders (e.g., Miller et al. 2009, Wang et al. 2010), and carabid beetles (Kavanaugh and Liang 2004 and 2006; Kavanaugh and Long 1999; Liang and Imura 2003; Liang and Kavanaugh 2006 and 2007; and Liu et al. 2010 and 2011).

Prior to the start of the project the carabid beetle fauna of the region was very poorly known. The faunal for the entire Hengduan region included only about 50 species (Yu 1992), and most of these were widespread species from low elevation areas. The region in general and the higher elevations in particular were virtually unexplored with respect to the carabid fauna. As a result of our work on this project to date, we now recognize more than 525 species occurring in the Gaoligong Shan, with many additional species undoubtedly represented among materials for groups not yet fully studied. For several of the groups currently under study, (e.g., *Leistus* (Nebriini), *Broscosom* a (Broscini), *Amerizus (Tiruka)* (Bembidiini) and *Aristochroa* (Pterostichini), species diversity is much higher in this area than is known anywhere else that these taxa occur.

This report, on the tribe Zabrini, represents the first of an intended series of treatments on the carabid beetle fauna of the Gaoligong Shan region, each dealing with one or more tribes or hyper-diverse genera represented in the fauna. These will appear as taxonomic work on each group is completed and not in any particular taxonomic or phylogenetic order.

Zabrini is a moderately diverse taxon, including nearly 700 described species (Lorenz 2005). It is principally Holarctic in distribution, with relatively few species occurring south of that region in the Neotropical (as far south as Costa Rica), Afrotropical (nine species in Ethiopia, Kenya, Somalia and Tansania) and Oriental (three or four species in northern parts only) Regions. The only zabrine genus represented in the study area is *Amara* Bonelli (1810), which is also the largest genus in the tribe, with just over 570 described species and a cumulative geographical range which actually defines that of the tribe. The ranges of the other zabrine genus, *Zabrus* Clairville (1806), and of *Pseudamara* Lindroth (1968), which has been transferred from the Zabrini to the Sphodrini recently by Hieke (2010, 2013), are fully within the range of *Amara*.

*Amara* is most diverse in temperate parts of the Holarctic Region. Along with members of the tribe Harpalini, zabrines are unusual among Carabidae in feeding mainly on seeds (Johnson and Cameron 1969), particularly those of the grass (Graminaceae) and mustard (Cruciferae) families, but also on fruits, flowers and other plant parts. Apparently, both adults and larvae use seeds as a main food source. Probably because of their feeding preferences, *Amara* species occur mainly in open areas, such as grasslands, meadows, forest edges, and disturbed habitats of all types, including both those associated with natural environmental processes (e.g., landslides, eroded or scoured stream banks, floodplains and areas burned by lightning strikes) and those created by humans (e.g., forest clearings, roadcuts and ruderal (waste) sites around human settlements or other constructs and agricultural sites).

As is the case with most other terrestrial anthropod groups, the *Amara* fauna of the study area has not been well documented previously. Most of our current knowledge of the Southeast Asian regional fauna is from the works of Baliani (1932, 1934a, 1934b, 1937, 1943), Hieke (see References section) and Jedlička (1934a, 1934b, 1956, 1957), with significant additional contributions from Andrewes (1930), Bates (1876, 1883, 1891), Morawitz (1863a, 1863b), Motschulsky (1844), Putzeys (1875) and Tschitschérine (1894, 1897, 1899). Based on our study of the material collected for the project and additional specimens from the region housed in other collections, we recognize a total of 13 *Amara* species found to occur in the study area, all of which have been described previously. We present here a key for identification of adults of these species, as well as nomenclatural data, diagnoses, illustrations of dorsal habitus and male genitalia, and information about geographical and habitat distributions within the study area and overall geographical distribution for each species. We also discuss geographical distributions of the species with respect to seven core areas and to each other, as well as broader geographical range patterns and the altitudinal ranges of the species.

## Materials and methods

**Figures 2–4.**
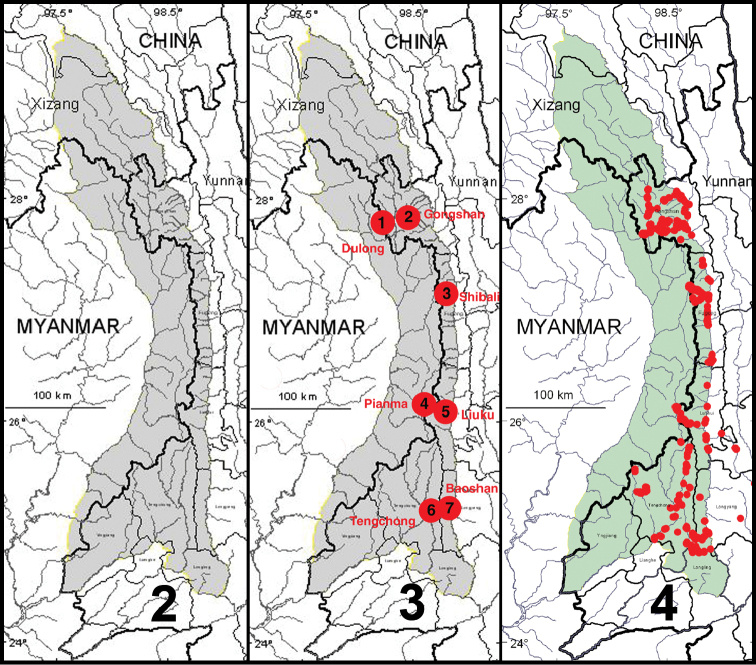
**2** Map showing natural extent of study area, colored in gray (however, sampling was permitted only in those portions in Yunnan Province **3** Map showing location of core sampling areas **4** Map showing locations of all entomological sampling sites.

The natural physiographic limits of the study area for the project are as shown in Fig. 2 and include areas in eastern Myanmar and southern Xizhang (Tibet); but we had permission to survey only those parts in Yunnan Province. Specialists for all taxonomic groups concentrated their efforts on seven core areas within the project region (Fig. 3), selected to facilitate comparisons of possible north to south and east to west spatial differences within the regional biota, as well as recognition of areas of local endemism. Other areas were sampled as time and opportunity permitted. The entomological team made a total of 13 expeditions to the Gaoligong region. Our sampling sites within the region are shown in Fig. 4. Habitats included in the study area range from subtropical lowland rainforest to the margin of glaciers and snowfields. In all, more than 35,000 carabid specimens were collected during the project by using a variety of collecting methods, including hand collecting both day and night, beating vegetation, sifting litter with subsequent extraction by hand or by mini-Winkler units, and Malaise flight traps and pitfall traps. All specimens were sorted to morphospecies (i.e., presumptive species units based on features of external structure and male and female genitalic traits) and detailed systematic studies of taxonomic groups are ongoing.

A total of 1,327 specimens representing zabrine species were collected during the project. All of these specimens have been divided among and are deposited in collections of our home institutions. Codens used throughout this report for collections in which specimens, including primary types, are deposited are as follows:

BMNH British Museum (Natural History), London, United Kingdom

CAS California Academy of Sciences, San Francisco, U.S.A.

CMEY Collection of P. Meyer, Darmstadt, Germany

CWRA Collection of D. Wrase, Berlin, Germany

DEI Deutsches Entomologisches Institut, Eberswalde, Germany

FRSDD Forest Research Institute, Dehra Dun, India

IOZ National Zoological Museum of China, Institute of Zoology, Beijing, China

IRSNB Institut Royal des Sciences Naturelles de Belgique, Brussels, Belgium

MCSNG Museo Civico di Storia Naturale, Genoa, Italy

MNHN Muséum National d’Histoire Naturelle, Paris, France

NHRS Naturhistoriska Riksmuseet, Stockholm, Sweden

NMPC National Museum (Natural History), Prague, Czech Republic

NZSI National Zoological Collection, Zoological Survey of India, Calcutta, India

ZIN Zoological Institute Academy of Sciences, St.Petersburg, Russia

ZFMK Zoologisches Forschungsmuseum “Alexander Koenig”, Bonn, Germany

ZMHB Museum für Naturkunde an der Humboldt-Universität, Berlin, Germany

ZMMU Zoological Museum, Moscow University, Moscow, Russia

The only measurements taken were of body length, measured from the anterior margin of the clypeus to the apex of the longer elytron. Digital images of dorsal habitus of a typical representative member of each morphospecies were taken using an Automontage imaging system from Synchroscopy with a JVC KY-F-75U digital camera and a Leica M420 dissecting microscope. The “CASENT” number associated with each image, as noted in figure captions, is a unique identifier that refers to the particular specimen photographed and its CAS database record. Distribution maps for each species were generated from geographical coordinate data maintained in a Biota Version 3.0 database (Colwell 2012) using the ArcMap program in ArcGIS for Desktop Version 10.2 software from Esri.

## Taxonomy

Adult specimens of species represented in the Gaoligong Shan region can be distinguished using the following key.

### Key for Identification of Adults of *Amara* species of the Gaoligong Shan region

**Table:** 

1	Medial protibial spurs trifid (Fig. 5a); dorsal surface dark metallic green, non-metallic black in a very few specimens; body length 7.0–8.5 mm; elytron with parascutellar pore-puncture present	*Amara (Zezea) davidi* Tschitschérine, 1897
–	Medial protibial spurs simple (Fig. 5b)	2
2	Last abdominal sternite of male with two pairs of setiferous punctures (Fig. 5d) near hind margin (second seta absent from one side in a few males for total of three setae); dorsal surface dark piceous; body length 7–8 mm; pronotum with both inner and outer basal impressions deep and broadly foveate (Fig. 14a)	*Amara (Xenocelia) sikkimensis* Andrewes, 1930
–	Last abdominal sternite of male with one pair of setiferous punctures (Fig. 5c) near hind margin (Fig. 5d)	3
3	Body length more than 11 mm; tarsomere 5 of hind tarsi with five or six setal pairs ventrally (Fig. 6a); dorsal surface dark piceous; body length 11–13 mm, body form stout; pronotum (Fig. 20a) with lateral margins markedly rounded, basal area punctate, in most specimens also punctate laterally in basal half and near anterior margin	*Amara (Bradytus) pingshiangi* Jedlička, 1957
–	Body length less; tarsomere 5 of hind tarsi with two (in a few specimens three) setal pairs ventrally (Fig. 6b)	4
4	Elytron with parascutellar pore-puncture present; dorsal surface with metallic luster	5
–	Elytra without parascutellar pore-puncture	7
5	Base of pronotum evenly convex from one side to the other, outer basal impressions absent or only very faintly suggested; body length 9–10 mm; sclerites of internal sac of median lobe of male aedeagus as in Fig. 7e–f	*Amara (Amara) congrua* Morawitz, 1863
–	Base of pronotum slightly flattened at the sides, only the middle part evenly convex, outer basal impressions evident, either shallow and obliquely linear (Fig. 11a) or deep and broadly foveate (Fig. 12a); sclerites of internal sac of median lobe of male aedeagus with different form (Fig. 7a–d)	6
6	Base of pronotum coarsely punctate; outer basal impressions shallow and obliquely linear (Fig. 11a); male aedeagus with median lobe distinctly broader in apical one-third than more basally, apical lamella shorter, broadly rounded apically and with sides only slightly convergent subapically (Fig. 11c); sclerites of internal sac of median lobe of male aedeagus as in Fig. 7a–b; body length 9–10 mm	*Amara (Amara) silvestrii* Baliani, 1937
–	Base of pronotum finely punctate; outer basal impressions deep and broadly foveate (Fig. 12a); male aedeagus with median lobe not or only slightly broader in apical one-third than more basally, apical lamella longer, narrowly rounded apically and with sides more distinctly convergent apically (Fig. 12c); sclerites of internal sac of median lobe of male aedeagus as in Fig. 7c–d; body length 8.5–10.0 mm	*Amara (Amara) shaanxiensis* Hieke, 2002
7	Dorsal surface light-brown to brownish black, without metallic reflection, entire legs and antennae pale; body length 6.5–8.0 mm; male aedeagus with apical third of median lobe broader than middle third (Fig. 21c), apical hook of right paramere large and slightly subapical (Fig. 8a)	*Amara (Reductocelia) lucidissima* Baliani, 1932
–	Dorsal surface darker, with or without distinct metallic reflection, at least femora dark (piceous to black)	8
8	Pronotum with lateral margins straight or faintly to distinctly sinuate just anterior to basal angles, rounded near middle, less rounded or nearly straight also in anterior one-third in most specimens, anterior angles distinctly and narrowly projected anteriorly beyond anterior margin; dorsal surface dark with distinct metallic blue-green reflection in most specimens, non-metallic black in a few specimens	9
–	Pronotum with lateral margins more or less evenly rounded from apical to basal angle, anterior angles not or only faintly and broadly projected anteriorly beyond anterior margin; dorsal surface with or without metallic reflection	10
9	Pronotum (Fig. 18a) with lateral explanation narrow throughout, outer basal impressions foveate and distinct from lateral groove, punctation of base not extended anteriorly along sides beyond basal one-third; elytral microsculpture effaced or nearly so in both males and females; body length 7.0–7.5 mm	*Amara (Bradytus) elegantula* Tschitschérine, 1899
–	Pronotum (Fig. 16a) with lateral explanation distinctly broader basally, outer basal impressions indistinct from lateral groove in most specimens, punctation of base extended anteriorly along sides to pronotal mid-length; elytral microsculpture comprised of isodiametric meshes, faintly impressed or nearly effaced in males, deeply impressed and distinct in females; body length 7.5–9.0 mm	*Amara (Bradytus) chalciope* (Bates, 1891)
10.	Elytral microscuplture comprised of distinctly transverse meshes in both males and females (more transverse and less deeply impressed in males than in females); pronotum (Fig. 19a) only slightly narrower anteriorly than basally, anterior margin almost as wide as posterior margin, pronotal base very coarsely punctate, outer basal impressions sharply delimited laterally by narrow, slightly oblique raised (but not carinate) areas	*Amara (Bradytus) simplicidens* Morawitz, 1863
–	Elytral microscuplture comprised of distinctly isodiametric meshes in both males and females (more deeply impressed in females than in males); pronotum (Figs 13a, 15a, 17a) more distinctly narrowed anteriorly than basally, anterior margin clearly narrower than posterior margin, pronotal base moderately coarsely punctate, outer basal impressions either not sharply delimited laterally by raised areas or, if so, then the raised area broader	11
11	Pronotum (Fig. 15a) with posterior angles distinctly rounded (narrowly so in some individuals), slightly to moderately obtuse; elytra with slight sub-basal depressions centered on striae 6 (visible on right elytron in Fig. 15a), also on striae 4 and/or 5 in some individuals; dorsal surface black, with very faint metallic blue or green metallic reflection in most individuals, more vivid (as in Fig. 15a) in or lacking from a few specimens; body length 8.5–9.0 mm	*Amara (Harpaloamara) latithorax* Baliani, 1934
–	Pronotum (Figs 13a, 17a) with posterior angles either obtusely angulate and slightly denticulate or narrowly rounded (if the latter, then body length 7.5 mm or less); elytra without evident sub-basal depressions; dorsal surface with or without metallic reflection	12
12	Dorsal surface with distinct metallic copper or bronze reflection, non-metallic black in very few specimens; pronotum (Fig. 13a) with posterior angles narrowly rounded, basal impressions deeply foveate but small in diameter, outer basal impressions not distinctly delimited laterally by a broad convexity; elytral striae distinct throughout but only shallowly impressed in most individuals; metatibiae of males without a brush-like patch of setae medially in the apical half; body length 6.5–7.5 mm	*Amara (Pseudoamara) birmana* Baliani, 1934
–	Dorsal surface black or piceous, without or with only very faint metallic green reflection; pronotum with posterior angles obtusely angulate and slightly denticulate, basal impressions broadly and deeply foveate, outer basal impression distinctly delimited laterally by a broad convexity; elytral striae distinct throughout and deeply impressed; metatibiae of males with a brush-like patch of setae medially in the apical half; body length 7.7–8.7 mm	*Amara (Bradytus) dissimilis* Tschitschérine, 1894

#### 1. Amara (Zezea) davidi

Tschitschérine, 1897

http://species-id.net/wiki/Amara_davidi

**Figure 5.**
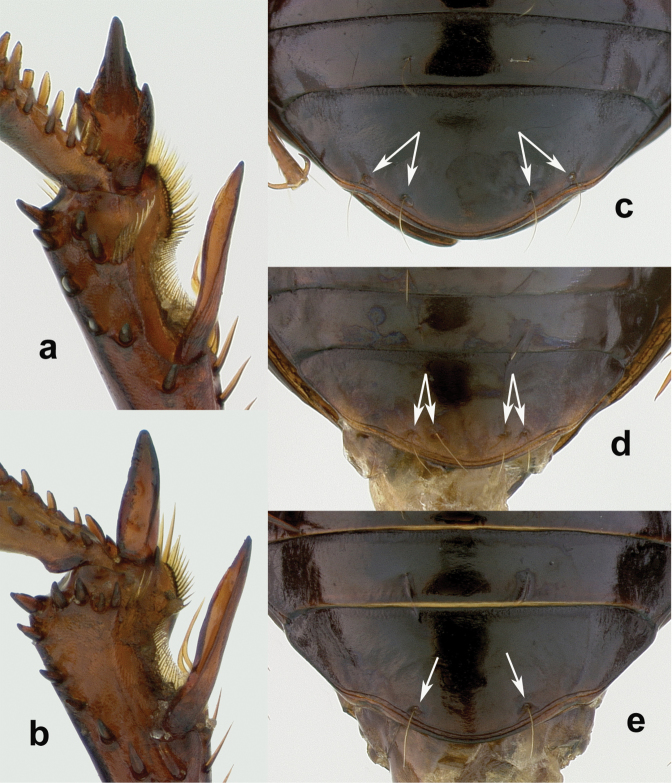
Tibial and abdominal apices. **a–b** right tibial apices, lateroventral aspect; a. *Amara davidi* Tschitshérine **b** *Amara sikkimensis* Andrewes, typical of all other *Amara* species of the area **c–e** abdominal apices, ventral aspect, with white arrows indicating insertion points for setae **c** *Amara sikkimensis*, typical of all *Amara* females **d** *Amara sikkimensis* Andrewes male **e** *Amara latithorax* Baliani, typical of males of all other *Amara* species of the area.

**Figure 6.**
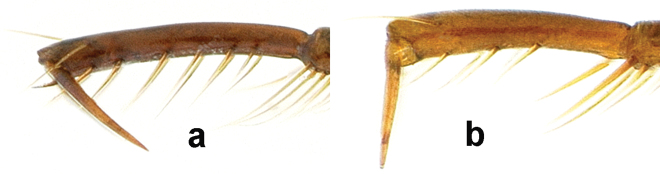
Tarsomere 5 of left hind tarsus, medial aspect. **a** *Amara pingshiangi* Jedlička **b** *Amara sikkimensis* Andrewes, typical of all other *Amara* species of the area.

Figs 5a, c, e , 6b , 9 , 22a , 27 [Fig F26] [Fig F27] –30

Amara (Triaena) davidi Tschitschérine, 1897: 67. Type material: Holotype male (“type”) and 1 paratype female in ZIN (Hieke 1970: 143), 4 paratypes in MNHN. Type locality: China, Sichuan, “Mou-Pin (leg. David)”.

##### Diagnosis.

Adults of this species (Fig. 9) can be distinguished from those of all other species in the region by their trifid medial protibial spurs (Fig. 5a). *Amara davidi* is the only member of subgenus *Zezea*, members of which share this feature, known from the region.

##### Habitat distribution.

Specimens of this species were collected from under stones in open roadside and waste areas (Fig. 22a) with scattered grasses and shrubs at elevations ranging from 2020 to 2440 m, syntopic (together in the same habitat) with adults of *Amara birmana*, *Amara shaanxiensis*, *Amara sikkimensis* and *Amara silvestrii* at one or more sites.

##### Geographical distribution within the Gaoligong Shan.

Fig. 9d. We examined a total of 3 specimens (all females) from the following localities: ***Gongshan County*:** Heiwadi (16.8 km W of Cikai on Dulong Valley Road, 27.79584°, 98.58443°, 2020 m, 20 April 2002, H.B. Liang, W.D. Ba, G.D. Yang & X.Q. Li collectors [2 females; CAS, IOZ]). ***Longyang County*:** Bawan-Tengchong Road Km 41 (near yakou, 24.93972°, 98.75333°, 2440 m, 15 October 2003, H.B. Liang & J.J. Yang (1 female; CAS).

Members of this species were collected in both the northern and southern parts of the study area (Core Areas 2, 6 and 7) but not in the central part. This gap in distribution is most likely an artifact of inadequate sampling and not a real disjunction.

##### Overall geographical distribution.

Fig. 27. This species has been recorded from Beijing, Gansu, Hubei, Qinghai, Shaanxi, Sichuan and Yunnan Provinces in China. Its occurrence in the study area represents the southwestern limit of its known geographical range.

#### 2. Amara (Amara) congrua

Morawitz, 1863

http://species-id.net/wiki/Amara_congrua

Figs 5b–c, e , 6b , 7e–f , 10 , 22b , 27 [Fig F26] [Fig F27] –30

Amara mongolica Motschulsky, 1844: 185, nomen oblitum. Type material: Number of syntypes not specified and unknown, a fragmentary male specimen in ZMMU (Keleinikova 1976: 206) identified as holotype in that collection by Hieke. Type locality: “sur la frontière de la Mongolie”, probably northeastern China, Outer Manchuria (now Primorsky Krai of Russia). Synonymy suggested by Morawitz 1863b: 63, synonymized by Hieke (1973: 116).
Amara (Amara) congrua Morawitz, 1863b: 62. Type material: Number of syntypes not specified, but at least several, with 5 syntypes in ZIN; lectotype male and 1 paralectotype designated by Hieke (1973: 25), the 3 other syntypes (paralectotypes) are adults of *Amara chalcites* Dejean. Type locality: Japan: Hakodate.
Amara zimmermanni Putzeys, 1875: LI (röm. 51) [nec Heer, 1837: 38]. Type material: Number of syntypes not specified, but at least several, with 3 syntypes in IRSNB and 2 in DEI (Döbler 1975: 148); two of those syntypes are adults of *Amara chalcites* Dejean, and the specimen from Kyoto in IRSNB, which has been considered as “Type”, is an adult of *Amara congrua* Morawitz. Type locality: Japan, Kyoto. Synonymized by Hieke (1973: 23), previously synonymized incorrectly with *Amara chalcites* Dejean by Bates (1876: 4).
Amara striatella Putzeys, 1875: LII (röm. 52). Type material: Number of syntypes not specified, but 4 of them in IRSNB; lectotype male designated by Hieke (1973: 27); one of those syntypes is an adult of *Amara chalcites* Dejean. Type locality: Japan, Kyoto. Synonymized by Hieke (1973: 23), previously synonymized incorrectly with *Amara chalcites* Dejean (as «var. *striatella*») by Bates (1883: 242).
Amara (Amara) mandzhurica Lutshnik, 1935: 257. Type material: Holotype female (not a male as stated by Lutshnik) in ZIN. Type locality: China: Manchuria: “Ertzendjantzy”. Synonymized by Hieke (1973: 58).
Amara (Amara) ovatoides Baliani, 1943: 38. Type material: Holotype female (not a male as stated by Baliani, but as can be seen in his Fig. 1), in MCSNG. Type locality: China, Shanghai. Synonymized by Hieke (1978: 314).
Amara (Amara) abnormalis Jedlička, 1956: 213. Type material: Holotype and 1 paratype in ZFMK, 2 paratypes in NMPC. Type locality: China, Fujian, Kuatun. Synonymized by Hieke (1973: 2).

##### Diagnosis.

**Figure 7.**
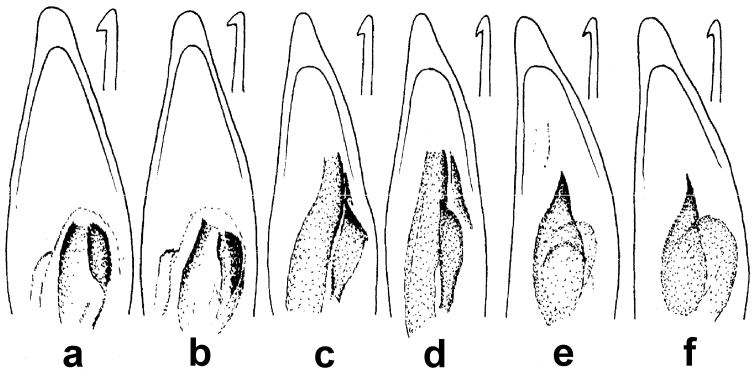
Apical and subapical region of median lobe (dorsal aspect) and apex of right paramere (medial aspect) of aedeagus of males. **a–b** *Amara silvestrii* Baliani **c–d** *Amara shaanxiensis* Hieke **e–f** *Amara congrua* Morawitz.

Adults of this species (Fig. 10) can be distinguished from those of all other species in the region by the following combination of character states: body length 9–10 mm; base of the pronotum evenly convex from one side to the other, outer basal impressions absent or only very faintly suggested; elytra with parascutellar pore puncture present; medial protibial spurs simple; tarsomere 5 of hind tarsi with two or (in a few specimens) three pairs of setae ventrally (Fig. 6b); last abdominal sternite of male with one pair (Fig. 5e) and female with two pairs (Fig. 5c) of setiferous punctures near hind margin; sclerites of internal sac of median lobe of male aedeagus with form as in Fig. 7e–f.

##### Habitat distribution.

Specimens of this species were collected in daytime from under stones and other cover in open roadside areas with scattered grasses and shrubs (Fig. 22b), at the edges of agricultural fields, including wet and dry rice paddies, and on open banks of streams, and in these same habitats at night, when beetles were found active on bare substrate. Specimens were found at elevations ranging from 892 to 2000 m. The only other *Amara* species members of which were found syntopic with those of *Amara congrua* was *Amara latithorax*, with adults of both species found together at only one site at an elevation of 1223 m.

##### Geographical distribution within the Gaoligong Shan.

Fig. 10d. We examined a total of 33 specimens (17 males and 16 females) from the following localities: ***Fugong County*:** Lumadeng Township (Chihengdi village, 26.98749°, 98.86310°, 1230 m, 29 April 2004, D.H. Kavanaugh & H.B. Liang collectors [3 males and 2 females; CAS, IOZ]); Shangpa Township (road on west side of Nu Jiang S of Shangpa, 26.88952°, 98.86539°, 1223 m, 22 April 2004, D.H. Kavanaugh & C.E. Griswold collectors [1 male and 1 female; CAS]). ***Gongshan County*:** Cikai Township (27.27064°, 98.66557°, 1500 m, 28 June 2000, D.H. Kavanaugh & H.B. Liang collectors [1 male; CAS]); Heiwadi village (27.78594°, 98.60117°, 1880-2000 m, 28 June 2000, D.H. Kavanaugh & H.B. Liang collectors [1 female; IOZ]). ***Lushui County*:** Liuku Township (Laimo village, 25.82767°, 98.85120°, 892 m, 15 May 2004, H.B. Liang & X.Y. Li collectors [1 male and 1 female; CAS, IOZ]). ***Tengchong County*:** Hehua Township (3.8 km S of Hehua along Daying Jiang at Nangyan village, 24.93873°, 98.38444°, 1140 m, 2 June 2006, D.H. Kavanaugh, R.L. Brett & D.Z. Dong collectors [1 female; CAS]), (5.4 km S of Hehua along Daying Jiang at Dengma village, 24.92346°, 98.38612°, 1105 m, 2 June 2006, D.H. Kavanaugh, R.L. Brett & D.Z. Dong collectors [1 female; IOZ]; Jietou Township (stream 0.7 km N of Jietou, 25.43128°, 98.64773°, 1564 m, 22 May 2006, D.H. Kavanaugh, R.L. Brett & H.B. Liang collectors [3 males and 2 females; CAS, IOZ]); Qingshui Township (Liangyang village in Rehai area, 24.94919°, 98.44921°, 1450 m, 1 June 2006, D.H. Kavanaugh, R.L. Brett, H.D. Liang, D.Z. Dong & P. Hu collectors [1 female; IOZ]); Qushi Township (Longchuan Jiang at Qinqiao in Qinqiao village, 25.27250°, 98.60083°, 1464 m, 6 June 2006, D.H. Kavanaugh, R.L. Brett & D.Z. Dong collectors [7 males and 5 females; CAS, IOZ, ZMHB]); Shangying Township (Bawan-Tengchong Road KM 63, 25.02917°, 98.66917°, 1360 m, 19 October 2003, H.B. Liang & X.C. Shi collectors [1 female; IOZ]); Wuhe Township (west bank of Longchuan Jiang at Tongjiazhuang village, 24.89284°, 98.67439°, 1210 m, H.B. Liang collector 24 May 2005 [1 male; IOZ]).

Members of this species were collected from the northern to the southern parts of the study area (Core Areas 2, 3, 5 and 6), but they were found only on the eastern side of the mountain range in northern and central areas (Core Areas 2,3 and 5) and only on the western versant in the southern part (Core Area 6). This distribution pattern is most likely an artifact of inadequate sampling on the western slope of the mountain range, much of which is in Myanmar.

##### Overall geographical distribution.

Fig. 27. This species has been recorded from China (Beijing, Fujian, Gansu, Guizhou, Hebei, Heilongjiang, Hongkong, Hubei, Jiangsu, Jiangxi, Jilin, Liaoning, Nei Mongol, Shaanxi, Shanghai, Sichuan, Yunnan and Zhejian Provinces), Japan (southern half), Laos, North and South Korea, Myanmar (extreme north), Russia (Primorskij Krai), Taiwan and Vietnam (extreme north).

#### 3. Amara (Amara) silvestrii

Baliani, 1937

http://species-id.net/wiki/Amara_silvestrii

Figs 5b–c , 5e , 6b , 7a–b , 11 , 23a , 27 [Fig F26] [Fig F27] –30

Amara (Amara) silvestrii Baliani, 1937: 179. Type material: Holotype male in MCSNG. Type locality: China, Yunnan “Yunnan-fu”.

##### Diagnosis.

Adults of this species (Fig. 11) can be distinguished from those of all other species in the region by the following combination of character states: body length 9–10 mm; base of pronotum slightly flattened at the sides, only the middle part evenly convex, coarsely punctuate, outer basal impressions shallow and obliquely linear (Fig. 11a); elytra with parascutellar pore puncture present; medial protibial spurs simple; tarsomere 5 of hind tarsi with two or (in a few specimens) three pairs of setae ventrally (Fig. 6b); last abdominal sternite of male with one pair (Fig. 5e) and female with two pairs (Fig. 5c) of setiferous punctures near hind margin; male aedeagus with median lobe distinctly broader in apical one-third than more basally, apical lamella shorter, broadly rounded apically and with sides only slightly convergent subapically (Fig. 11c), sclerites of internal sac with form as in Fig. 7a–b.

##### Habitat distribution.

Specimens of this species were collected in daytime from under stones and other cover in open roadside areas (Fig. 23a) and meadows with scattered grasses and shrubs, at the edges of agricultural fields, including wet and dry rice paddies, under clods of soil in recently tilled fields and on open banks of streams, and in these same habitats at night, when beetles were found active on bare substrate. They were also collected under debris and in leaf litter in deciduous forests and also in crevices between stones in a talus slope. Members of this species were found at elevations ranging from 1515 to 3000 m, syntopic with adults of *Amara birmana*, *Amara chalciope*, *Amara davidi*, *Amara lucidissima*, *Amara shaanxiensis*, and *Amara sikkimensis* at one or more sites.

##### Geographical distribution within the Gaoligong Shan.

Fig. 11d. We examined a total of 34 specimens (18 males and 16 females) from the following localities: ***Fugong County*:** Lishadi Township (Shibali area, 27.16536°, 98.78003°, 2535 m, D.H. Kavanaugh, P. Paquin & D.Z. Dong collectors [1 female; CAS]), (0.5 km W of Shibali, 27.16665°, 98.77936°, 2537 m, 18 August 2005, P. Paquin collector [1 male and 1 female; CAS]), (below Shibali on Yaping Road, 27.16520°, 98.77980°, 2530 m, 24 April 2004m, H.B. Liang & X.Y. Li collectors [1 male; IOZ]), (7.5 km below Shibali on Yaping Road, 27.14627°, 98.81559°, 2030 m, 3 May 2004, H.B. Liang & M. Xi collectors [1 female; IOZ]); Lumadeng Township (0.5 km W of Lao Shibali on Lao Shibali Road, 27.08072°, 98.76920°, 2305 m, 22 August 2008, P. Paquin collector [1 male; CAS]), (South Fork of Yamu He at Yejiadi, 27.08994°, 98.77325°, 2307 m, 10 May 2004, H.B. Liang collector [2 males and 1 female; CAS, IOZ); Maji Township (Majimi village near power station on Gaxie He, 27.39630°, 98.81701°, 1567 m, 28 April, 2004, H.B. Liang collector [1 male; IOZ]). ***Gongshan County*:** Cikai (27.74972°, 98.66444°, 1515 m, 5 October 2002, H.B. Liang & W.D. Ba [1 female; IOZ]); Dabadi (41 km W of Cikai on Dulong Valley Road, 27.79655°, 98.50562°, 3000 m, 27 September to 6 October 2002, D.H. Kavanaugh, P.E. Marek, H.B. Liang & D.Z. Dong collectors [5 males and 6 females; CAS, IOZ, ZMHB]); Heiwadi (16.8 km W of Cikai on Dulong Valley Road, 27.79584°, 98.58443°, 2020 m, 15 and 20 April 2002, H.B. Liang, W.D. Ba & C.G. Jin collectors [2 males and 1 female; IOZ, ZMHB]); Qiqi area (27.71542°, 98.56529°, 2000-2020 m, 9-14 July 2000, D.H. Kavanaugh & H.B. Liang collectors [1 female; CAS]); Cikai Township (8.3 to 13.1 km NW of Cikai on Dulong Valley Road, 27.75653°, 98.58214°, 2620-3000 m, 23 September 2002, D.H. Kavanaugh, P.E. Marek & D.Z. Dong collectors [1 female; CAS]). ***Lushui County*:** Luzhang Township (Yaojiaping He at Pianma Road, 25.97722°, 98.71091°, 2527 m, 20 May 2005, D.H. Kavanaugh, C.E. Griwold, H.B. Liang, D.Z. Dong & G. Tang collectors [1 male; CAS]); Pianma Township (6 km ESE of Pianma, 26.00808°, 98.65921°, 2210 m, 15 May 2005, H.B. Liang & D.Z. Dong collectors [2 males and 2 females; CAS, IOZ]). ***Tengchong County*:** Jietou Township (Cha He at Shaba village, 25.39256°, 98.70488°, 1840 m, 25 May 2006, D.H. Kavanaugh, R.L. Brett & D.Z. Dong collectors [1 male; CAS]); Qushi Township (Longchuan Jiang at Longkou village, 25.28167°, 98.59167°, 1500 m, D.H. Kavanaugh & C.E. Griswold collectors [1 male; CAS]).

Members of this species were collected from the northern to the southern parts of the study area (Core Areas 2, 3, 5 and 6), but they were found only on the eastern side of the mountain range in northern half of the study area (Core Areas 2 and 3), on both side in the central part (Core Areas 3 and 4) and only on the western versant in the southern part (Core Area 6). This distribution pattern is most likely an artifact of inadequate sampling on the western slope of the mountain range in the north, some of which is in Myanmar.

##### Overall geographical distribution.

Fig. 27. This species has been recorded from China (Gansu, Hubei, Shaanxi, Sichuan and Yunnan Provinces), Myanmar (extreme north) and Taiwan. Its occurrence in the study area represents the southwestern limit of its known geographical range.

#### 4. Amara (Amara) shaanxiensis

Hieke, 2002

http://species-id.net/wiki/Amara_shaanxiensis

Figs 5b–c , 5e , 6b , 7c–d , 12 , 22a , 25b , 27 [Fig F26] [Fig F27] –30

Amara (Amara) shaanxiensis Hieke, 2002: 663. Type material: Holotype male and 1 paratype in CWRA, 2 male and 2 female paratypes in ZMHB. Type locality: China, Shaanxi, Zhouzi Xian, Pass between Banfangzi and Xingian, 2000 m.

##### Diagnosis.

Adults of this species (Fig. 12) can be distinguished from those of all other species in the region by the following combination of character states: body length 8.5–10 mm; base of pronotum slightly flattened at the sides, only the middle part evenly convex, finely punctate; outer basal impressions deep and broadly foveate (Fig. 12a); elytra with parascutellar pore puncture present; medial protibial spurs simple; tarsomere 5 of hind tarsi with two or (in a few specimens) three pairs of setae ventrally (Fig. 6b); last abdominal sternite of male with one pair (Fig. 5e) and female with two pairs (Fig. 5c) of setiferous punctures near hind margin; male aedeagus with median lobe not or only slightly broader in apical one-third than more basally, apical lamella longer, narrowly rounded apically and with sides more distinctly convergent apically (Fig. 12c), sclerites of internal sac with form as in Fig. 7c-d.

##### Habitat distribution.

Specimens of this species were collected in daytime from under stones and other cover in open roadside areas (Figs 22a, 25b), meadows and marshy areas with scattered grasses and shrubs, at the edges of agricultural fields, including wet and dry rice paddies and on open banks of streams, and in these same habitats at night, when beetles were found active on bare substrate. They were also collected under debris in daytime and on the surface of leaf litter at night in deciduous forests. Members of this species were found at elevations ranging from 1837 to 3000 m, syntopic with adults of *Amara birmana*, *Amara chalciope*, *Amara davidi*, *Amara lucidissima*, *Amara pingxiangi*, *Amara sikkimensis* and *Amara silvestrii* at one or more sites.

##### Geographical distribution within the Gaoligong Shan.

Fig. 12d. We examined a total of 88 specimens (36 males and 52 females) from the following localities: ***Gongshan County*:** Dabadi (41 km W of Cikai on Dulong Valley Road, 27.79655°, 98.50562°, 3000 m, D.H. Kavanaugh, P.E. Marek, H.B. Liang, D.Z. Dong & X.C. Liang collector [1 male and 4 females; CAS, IOZ]). ***Longling County*:** Xiaheishan Forest Reserve (Guchengshan at 1.2 km SSE of Km 23.5 on Route 23.5, 24.82888°, 98.76001°, 2020 m, 25-26 May 2005, D.H. Kavanaugh, C.E. Griswold, H.B.Liang and D.Z. Dong collectors [4 males and 4 female; CAS]). ***Longyang County*:** Bawan-Tengchong Road (Km 29-35, 24.92916°, 98.75861°, 2000-2350 m, 12 October 2003, D.Z. Dong collector [1 female; IOZ]), (Km 36-37, 24.93417°, 98.77944°, 2150 m, H.B. Liang & X.C. Shi collectors [2 males and 1 female; IOZ, ZMHB]), (Km 40–41, 24.92694°, 98.75278°, 2404 m, 12 October 2003, H.B. Liang & X.C. Shi collectors [1 female; IOZ]), (Km 41 near yakou, 24.93972°, 98.75333°, 2440 m, 15 October 2003, H.B. Liang & J.J. Yang collectors [1; female; IOZ]), (Km 42 at Sanchawa, 24.94750°, 98.75556°, 2300 m, 13 October 2003, H.B. Liang & X.C. Shi collectors [1 male; CAS]; Luoshuidong area (at Sancha He, 24.94833°, 98.75667°, 2300 m, 30 May 2005, D.H. Kavanaugh & H.M. Yan collectors [1 female; CAS]; 3 June 2005, D.H. Kavanaugh, C.E. Griswold, H.B. Liang, D.Z. Dong & H.M Yan collectors [1 male; CAS]); Nankang Forestry Station (at Km 19.8 on Route S317, 24.82284°, 98.78207°, 2060 m, 23 May 2005, D.H. Kavanaugh, C.E. Griswold, H.B. Liang, D.Z. Dong, H.M. Yang & G. Tang collectors [3 males and 7 females; CAS, IOZ, ZMHB]; Nakang Yakou (24.82587°, 98.76832°, 2148 m, 22 May 2005, H.B. Liang collector [(just N of yakou, 24.83178°, 98.76472°, 2180 m, 22 May 2005, D.H. Kavanaugh, C.E. Griswold & D.Z. Dong collectors [2 males and 2 females; CAS, IOZ]; 25 May 2005, D.H. Kavanaugh & C.E. Griswold [1 female; CAS]). ***Lushui County*:** Luzhang Township (Lusai He, 25.96378°, 98.77032°, 1873 m, 20 May 2005, H.B. Liang & D.Z. Dong collectors [5 males; CAS, IOZ]). ***Tengchong County*:** Houqiao Township (5.9 airkm NE of Houqiao below Guyong Forestry Station, 25.36562°, 98.31610°, 2030 m, 27 May 2006, D.H. Kavanaugh, R.L. Brett, H.B. Long, D.Z. Dong & Z.C. Liu collectors [1 male and 3 females; CAS, IOZ]); Jietou Township (2.0 km N of Dahetou Ligganjiao on Longtang He, 25.75743°, 98.69457°, 2080 m, 16 May 2006, D.Z. Dong collector [1 male; CAS]), (1.4 km S of Dahetou Ligganjiao along Longchuan Jiang, 25.72717°, 98.69322°, 1960 m, 16 May 2006, H.B. Liang collector [2 males and 3 females; CAS, IOZ]), (Longtang He at Dahetou Lingganjiao, 25.73947°, 98.69630°, 2010 m, 14-20 May 2006, D.H. Kavanaugh, R.L. Brett, H.B. Liang & P. Hu collectors [8 males and 15 female; CAS, IOZ]); Shangying Township (Bawan-Tenchong Road Km 42-46, 24.95361°, 98.74222°, 2290 m, 14 and 17 October 2003, H.B. Liang & X.C. Shi collectors [4 males and 2 females; CAS, IOZ]).

Members of this species were collected in both the northern and southern parts of the study area (Core Areas 2, 5, 6 and 7), but not in the central part (Core Area 3). This gap in distribution is most likely an artifact of inadequate sampling and not a real disjunction.

##### Overall geographical distribution.

Fig. 27. This species has been recorded from Shaanxi and Yunnan Provinces in China, and its occurrence in the study area represents the southwestern limit of its known geographical range.

#### 5. Amara (Pseudoamara) birmana

Baliani, 1934

http://species-id.net/wiki/Amara_birmana

Figs 5b–c , 5e , 6b , 13 , 22a , 23a , 25a , 25b , 27 [Fig F26] [Fig F27] –30

Amara (Amara) birmana Baliani, 1934a: 189. Type material: Holotype female in BMNH, 1 paratype female in MCSNG. Type locality: Burma [without specific locality, but probably from the mountains of northern Myanmar near the border with Yunnan Province, China]. Transferred to subgenus *Pseudoamara* Baliani by Hieke (2002: 624).
Amara (Celia) yunnana Baliani, 1934a: 193. Type material: Holotype male and 6 paratypes in BMNH. Type locality: China, Yunnan, Yunnan-fou. Synonymized by Hieke (1975: 333).
Amara (Pseudoamara) beesoni Baliani, 1934a: 190. Type material: Holotype male and allotype in BMNH, 4 paratypes in BMNH and MCSNG. Type locality: India, Assam, Shillong. Synonymized by Hieke (1975: 285).

##### Diagnosis.

**Figure 8.**
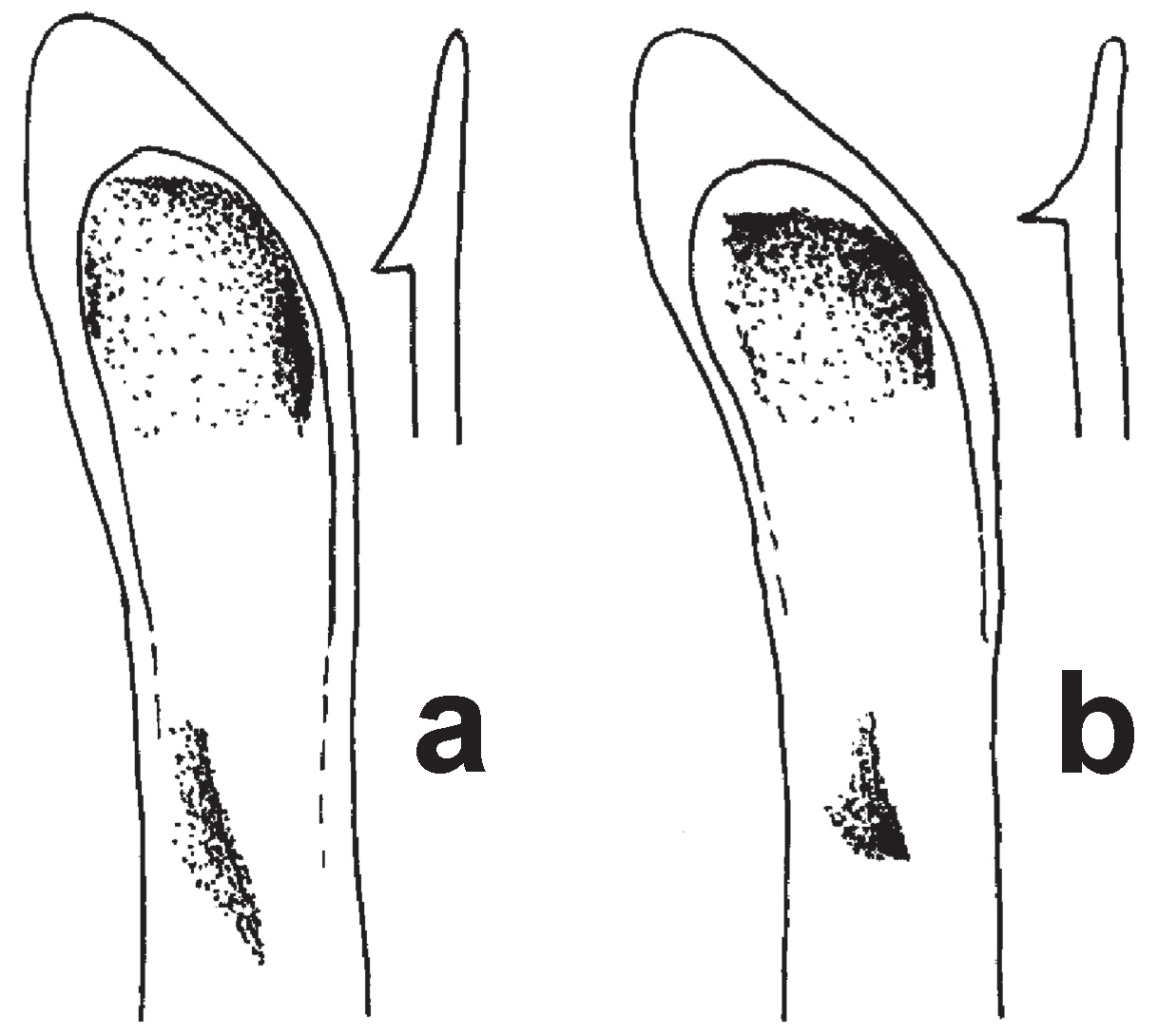
Apical region of median lobe (dorsal aspect) and apex of right paramere (medial aspect) of aedeagus of males. **a** *Amara lucidissima* Baliani **b** all other species of subgenus *Reductocelia* (for comparative purposes only, no other species of this subgenus in the area).

Adults of this species (Fig. 13) can be distinguished from those of all other species in the region by the following combination of character states: body length 6.5-7.5 mm; dorsal surface with distinct metallic copper or bronze reflection, non-metallic black in very few specimens, at least femora and outer antennomeres dark (piceous to black); elytral microscuplture comprised of distinctly isodiametric meshes in both males and females (more deeply impressed in females than in males); pronotum (Fig. 13a) more distinctly narrowed anteriorly than basally, anterior margin clearly narrower than posterior margin, lateral margins more or less evenly rounded from apical to basal angle, anterior angles not or only faintly and broadly projected anteriorly beyond anterior margin, posterior angles narrowly rounded, basal impressions deeply foveate but small in diameter, outer basal impressions not distinctly delimited laterally by a broad convexity; elytra without evident sub-basal depressions, parascutellar pore puncture absent, elytral striae distinct throughout but only shallowly impressed in most individuals; medial protibial spurs simple; metatibiae of males without a brush-like patch of setae medially in the apical half; tarsomere 5 of hind tarsi with two or (in a few specimens) three pairs of setae ventrally (Fig. 6b); last abdominal sternite of male with one pair (Fig. 5e) and female with two pairs (Fig. 5c) of setiferous punctures near hind margin; male aedeagus with apical third of median lobe about as broad as middle third, apical hook of right paramere smaller and closer to apex (Fig. 8a).

**Figure 9.**
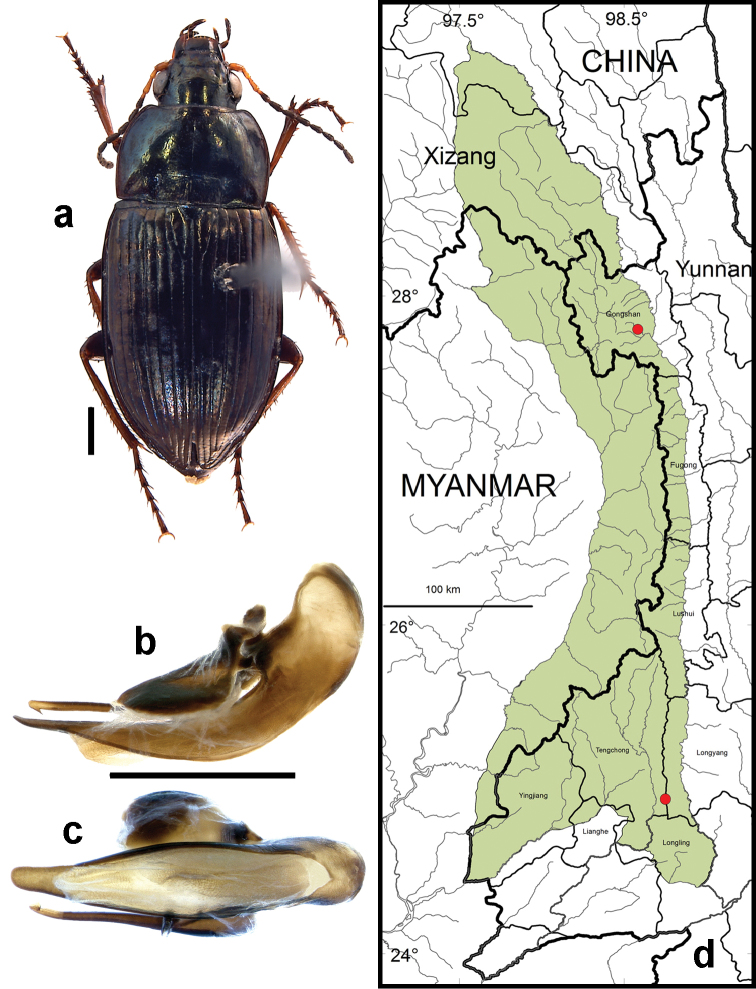
*Amara (Zezea) davidi* Tschitschérine. **a** dorsal habitus (CASENT1010925) **b–c** median lobe of aedeagus of male (CASENT8125447) **b** left lateral aspect **c** dorsal aspect; scale lines = 1.0 mm **d** Map of localities records (red circles) for *Amara davidi* in the Gaoligong Shan region, scale line = 100 km.

**Figure 10.**
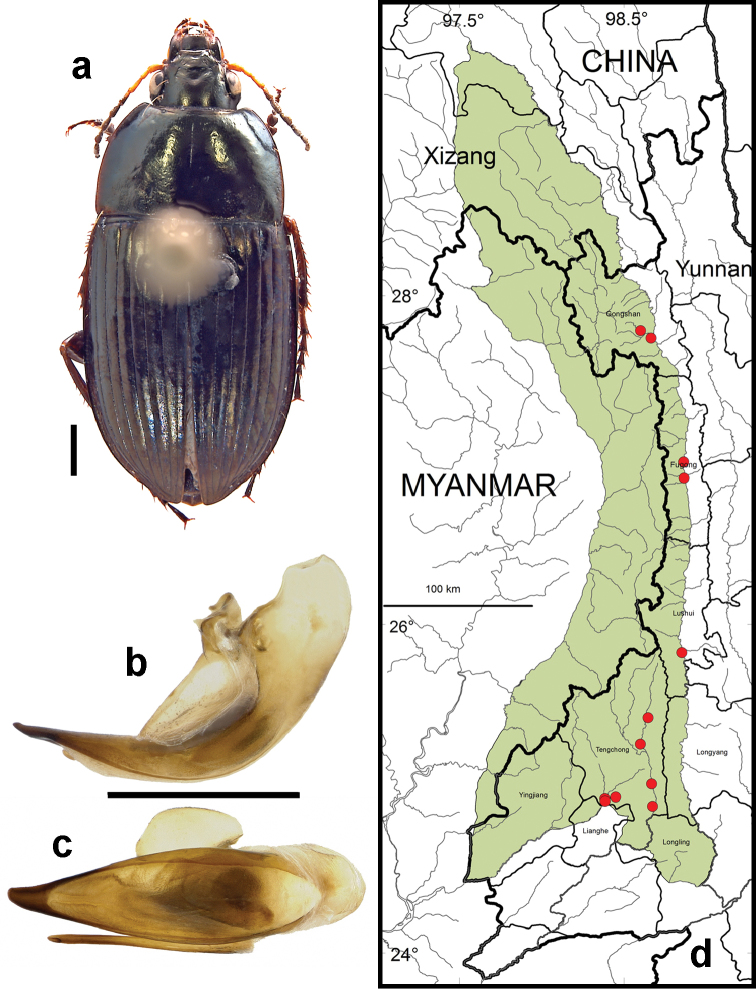
*Amara (Amara) congrua* Morawitz. **a** dorsal habitus (CASENT1039647) **b–c** median lobe of aedeagus of male (CASENT1039647) **b** left lateral aspect **c** dorsal aspect; scale lines = 1.0 mm **d** Map of localities records (red circles) for *Amara congrua* in the Gaoligong Shan region, scale line = 100 km.

**Figure 11.**
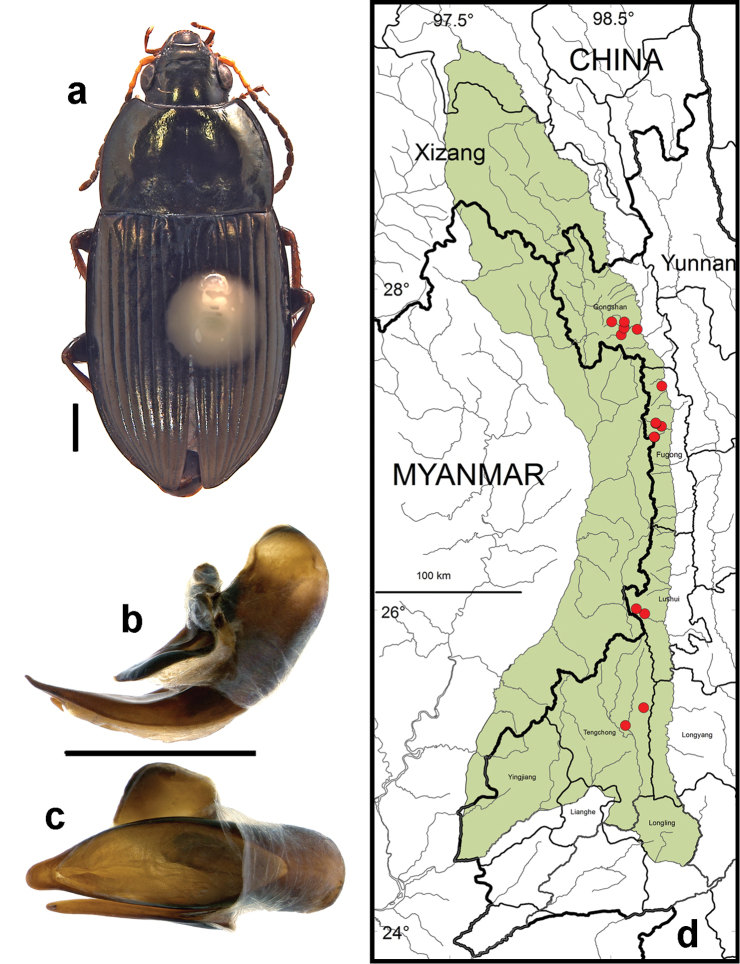
*Amara (Amara) silvestrii* Baliani. **a** dorsal habitus (CASENT1035225) **b–c** median lobe of aedeagus of male (CASENT1035225) **b** left lateral aspect **c** dorsal aspect; scale lines = 1.0 mm **d** Map of localities records (red circles) for *Amara silvestrii* in the Gaoligong Shan region, scale line = 100 km.

**Figure 12.**
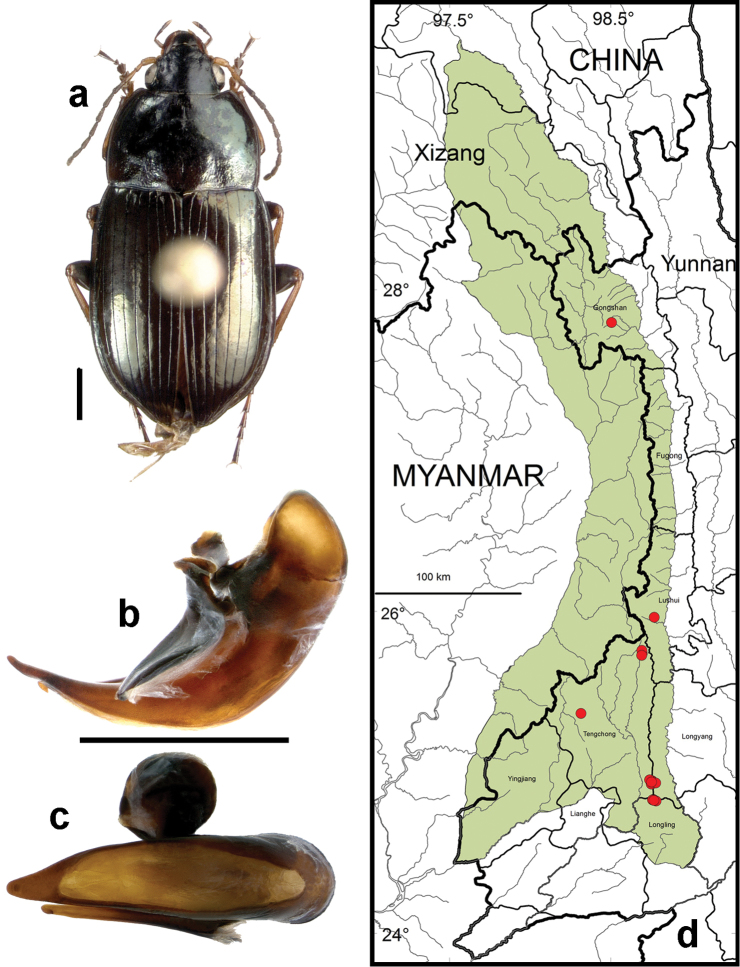
*Amara (Amara) shaanxiensis* Hieke. **a** dorsal habitus (CASENT1013215) **b–c** median lobe of aedeagus of male (CASENT1024088) **b** left lateral aspect **c** dorsal aspect; scale lines = 1.0 mm **d** Map of localities records (red circles) for *Amara shaanxiensis* in the Gaoligong Shan region, scale line = 100 km.

**Figure 13.**
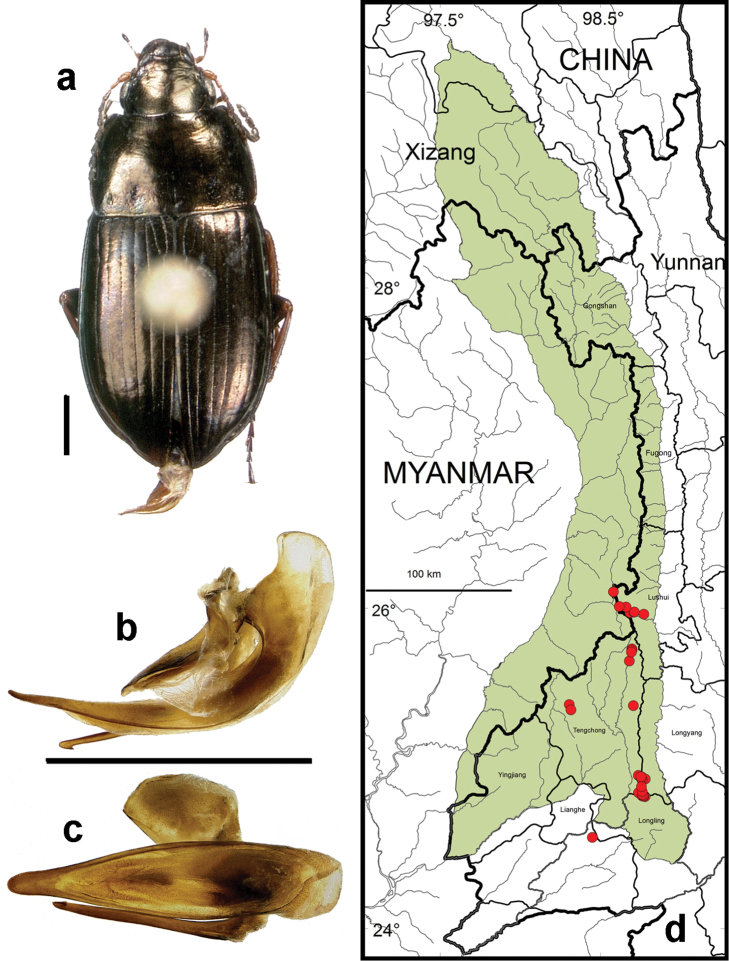
*Amara (Pseudoamara) birmana* Baliani. **a** dorsal habitus (CASENT1011859) **b–c** median lobe of aedeagus of male (CASENT1039066) **b** left lateral aspect **c** dorsal aspect; scale lines = 1.0 mm **d** Map of localities records (red circles) for *Amara birmana* in the Gaoligong Shan region, scale line = 100 km.

##### Habitat distribution.

Specimens of this species were collected, often in great abundance, in daytime from under stones and other cover in open roadside areas (Figs 22a, 25a) and waste areas around human settlements (Figs 23a, 25b) with scattered grasses and shrubs, at the edges of agricultural fields and on open banks of streams, and in these same habitats at night, when beetles were found active on bare substrate. They were also collected under rotting logs on a deciduous forest floor. Members of this species were found at elevations ranging from 1590 to 3150 m, syntopic with adults of *Amara chalciope*, *Amara davidi*, *Amara dissimilis*, *Amara lucidissima*, *Amara pingxiangi*, *Amara shaanxiensis*, *Amara sikkimensis* and *Amara silvestrii* at one or more sites.

##### Geographical distribution within the Gaoligong Shan.

Fig. 13d. We examined a total of 244 specimens (126 males and 118 females) from the following localities: ***Longling County*:** Longjiang Township (small stream along road at 1.2 km SSE of Km 23.5 on Route 23.5, 24.82888°, 98.76001°, 2020 m, 25–27 May 2005, D.H. Kavanaugh, C.E. Griswold, H.B. Liang & D.Z. Dong collectors [4 males and 5 females; CAS, IOZ]), (Xiaoheishan Forest Station, 24.83671°, 98.76185°, 2067 m, 28 May 2005, H.B. Liang, K.J. Guo & H.M. Yan collectors [2 females; CAS, IOZ]). ***Longyang County*:** Bawan Township (mountain near Nankang Yakou, 24.83250°, 98.76944°, 2245 m, 27 October 2003, H.B. Liang & X.C. Shi collectors [3 males and 5 females; CAS, IOZ]), (Km 24 on Baoshan-Tengchong Highway near Nankang Yakou, 24.82583°, 98.77222°, 2130 m, 26 October 2003, H.B. Liang & X.C. Shi collectors [6 males and 5 females; CAS, IOZ]), (Km 36–37 on Bawan-Tenchong Road, 24.93417°, 98.77944°, 2150 m, 12 October 2003, H.B. Liang & X.C. Shi collectors [1 female; IOZ]), (Km 40 on Bawan-Tenchong Road at Dasheyao Plant Protection Station, 24.92944°, 98.75861°, 2320 m, 16 October 2003, H.B. Liang & J.J Yang collectors [1 female; IOZ], 3 June 2005, D.H. Kavanaugh, D.Z. Dong & J.J. Yang collectors [1 male and 1 female; CAS, IOZ]), 90 km W of Baoshan, 26–28 May 1995, S. Bečvar collector [1 female; ZMHB]), (Luoshuidong area at Sancha He, 24.94833°, 98.75667°, 2300 m, 26–31 October 1998, D.H. Kavanaugh & C.E. Griswold collectors [6 males and 4 females; CAS, IOZ, ZMHB], 3 June 2005, D.H. Kavanaugh, C.E. Griswold, H.B. Liang, D.Z. Dong & H.M. Yan collectors [1 female; CAS], 24.94865 0176/98.75193°, 2350 m, 30 May 2005, H.B. Liang & J.J. Yang collectors [3 males and 1 female; CAS, IOZ]), (Nankang Forest Station (24.82444°, 98.77889°, 2085 m, 27 October 2003, H.B. Liang & X.C. Shi collectors [9 males and 12 females; CAS, IOZ]), (Nankang Yakou (24.83167°, 98.76667°, 2130 m, 4–7 November1998, D. H. Kavanaugh collector [25 males and 20 females; CAS, IOZ, ZMHB], 24.82587°, 98.76832°, 2148 m, 22 May 2005, H.B. Liang collector [1 male and 1 female; CAS, IOZ], 24.83124°, 98.76843°, 2210 m, 23 May 2005, H.B. Liang collector [2 females; CAS, IOZ]), (just N of Nankang Yakou, 24.83178°, 98.76472°, 2180 m, 22 and 26 May 2005, H.B. Liang & D.Z. Dong collectors [5 males and 4 females; CAS, IOZ]). ***Lushui County*:** Luzhang Township (Km 44.7 on Pianma Road at Yaojiaping, 25.97538°, 98.71006°, 2516 m, 11 May 2005, D.H. Kavanaugh, H.B. Liang, D.Z. Dong & G. Tang collectors [1 female; CAS]), (Fengxue Yakou at Pianma Road, 25.97228°, 98.68336°, 3150 m, D.H. Kavanaugh, P.E. Marek & H.B. Liang collectors [1 female; CAS]), (Lusai He, 25.96378°, 98.77032°, 1873 m, 20 May 2005, H.B. Liang & D.Z. Dong collectors [3 amles and 1 female; CAS, IOZ]), (Yaojiaping He at Pianma Road, 25.97722°, 98.71091°, 2527 m, 20 May 2005, D.H. Kavanaugh, C.E. Griswold, H.B. Liang, D.Z. Dong & G. Tang collectors [1 female; IOZ]); Pianma Township (6 km ESE of Pianma, 26.00808°, 98.65921°, 2310 m, 15 May 2005, H.B. Liang & D.Z. Dong collectors [5 males and 6 females; CAS, IOZ]), (20 km N of Pianma along Gangfang He at Gulang Village, 26.10321°, 98.58094°, 1590 m, 14 May 2005, H.B. Liang collector [1 female; IOZ]), (Xia Pianma Village, 26.01137°, 98.61788°, 1850 m, 13 May 2005, H.B. Liang collector [1 male; IOZ]). ***Tengchong County*:** Houqiao Township (5.9 airkm NE of Houqiao below Guyong Forestry Station, 25.3562°, 98.31610°, 2030 m, 27 May 2006, D.H. Kavanaugh, R.L. Brett & D.Z. Dong collectors [1 female; IOZ]), (8.5 airkm NNE of Houqiao at Gaoshidong, 25.39858°, 98.30533°, 2580 m, 27 May 2006, D.H. Kavanaugh, R.L. Brett & D.Z. Dong collectors [1 male and 1 female; CAS, IOZ]); Jietou Township (Dahetou Lingganjiao, Longtang He, 25.73947°, 98.69630°, 2010 m, 14–16 and 18 May 2006, D.H. Kavanaugh, R.L. Brett & H.B. Liang collectors [16 males and 11 female; CAS, IOZ], 0.3 km S in Longchuan Jiang valley, 25.73678°, 98.69639°, 2005 m, 18 May 2006, D.Z. Dong collector [4 males and 5 females; CAS, IOZ], 1.4 km S along Longchuan Jiang, 25.72717°, 98.69322°, 1960 m, 19 May 2006, D.H. Kavanaugh, R.L. Brett & D.Z. Dong collectors [2 females; CAS, IOZ], 0.75 km N on Longtang He, 25.74622°, 98.69612°, 2030 m, 18 May 2006, D.H. Kavanaugh & R.L. Brett collectors [1 female; CAS]), (Longchuan Jiang from Dahetou Village to Dahetou Lingganjiao, 25.67125°, 98.68016°, 1838–2010 m, 14 May 2006, H.B. Liang collector [2 males and 1 female; CAS, IOZ]), (Shaba Village, Cha He, 25.39256°, 98.70488°, 1840 m, 25 May 2006, D.H. Kavanaugh, R.L. Brett & D.Z. Dong collectors [1 male and 1 female; CAS]); Shangying Township (Km 41 on Bawan-Tenchong Road near Yakou, 24.93972°, 98.75333°, 2440 m, 15 October 2003, H.B. Liang, X.C. Shi & D.Z. Dong collectors [2 males and 1 female; CAS, IOZ]), (Km 42–46 on Bawan-Tenchong Road, 24.95361°, 98.74222°, 2290 m, 14 and 17 October 2003, H.B. Liang & X.C. Shi collectors [7 males and 2 female; CAS, IOZ]), (Km 46–51 on Bawan-Tenchong Road, 24.95722°, 98.73667°, 2220 m, 17 October 2003, H.B. Liang & X.C. Shi collectors [1 male; IOZ]); Wuhe Township (Km 24 on Baoshan-Tengchong Highway, 24.82889°, 98.76028°, 2008 m, 29 October 2003, N.D. Penny, T. Briggs & X.Y. Li collectors [6 males; CAS, IOZ]), (Km 28.8 on Route S317 at Zhengding Forestry Station, 24.84855°, 98.73761°, 1834 m, 23 May 2005, D.Z. Dong & H.M. Yan collectors [1 male and 2 females; CAS, IOZ]), (31 km SE of Tengchong, 24.88639°, 98.75611°, 26 August 2009, D.W. Wrase collector [1 male and 2 females; ZMBH]), (33 km SE of Tengchong, 24.85611°, 98.76000°, 2100–2200 m, 31 May and 4 June 2007, A. Pütz collector [2 males and 1 female; ZMHB], 31 May 2007, D.W. Wrase collector [1 male; ZMHB]), (100 km W of Baoshan, 14–21 June 1993, E. Jendek & O. Sausa collectors [1 male; ZMHB]), (Xiaodifang Village, 24.85722°, 98.75917°, 2150 m, 29 October 2003, D.Z. Dong collector [3 males and 1 female; CAS, IOZ]), (Xiaoheishan Forest Station, 24.82889°, 98.76000°, 2025 m, 29 October 2003, H.B. Liang collector [6 males and 7 females; CAS, IOZ]).

Members of this species were collected only in the southern half of the study area (Core Areas 4, 5, 6 and 7), on both eastern and western slopes of the mountain range.

##### Overall geographical distribution.

Fig. 27. This species has been recorded from China (Sichuan and Yunnan Provinces), India (Assam, Arunashal Pradesh and Sikkim) and Myanmar. Its occurrence in the study area represents the southern limit of its known geographical range.

#### 6. Amara (Xenocelia) sikkimensis

Andrewes, 1930

http://species-id.net/wiki/Amara_sikkimensis

**Figure 14.**
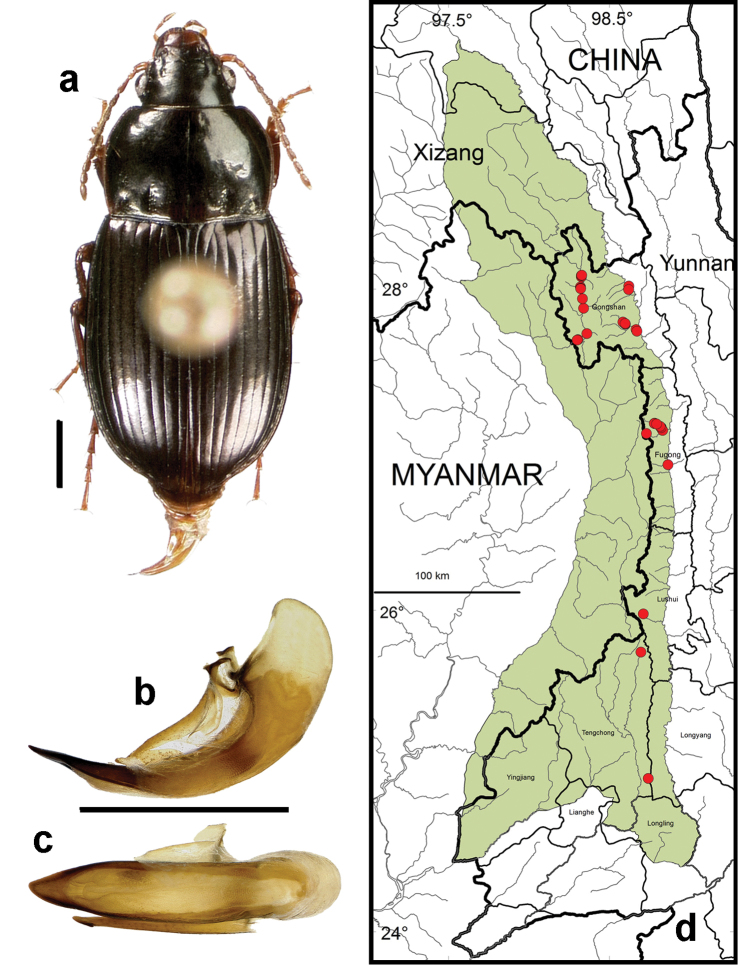
*Amara (Xenocelia) sikkimensis* Andrewes. **a** dorsal habitus (CASENT1015288) **b–c** median lobe of aedeagus of male (CASENT1025342) **b** left lateral aspect **c** dorsal aspect; scale lines = 1.0 mm **d** Map of localities records (red circles) for *Amara sikkimensis* in the Gaoligong Shan region, scale line = 100 km.

Figs 5b–d , 6b , 14 , 23a , 23b , 27 [Fig F26] [Fig F27] –30

Amara (Leiocnemis) sikkimensis Andrewes, 1930: 24. Type material: Holotype male and 7 paratypes in BMNH. Type locality: China, Xizang Autonomous Region (southern Tibet), Rongshar Valley. Transferred to subgenus *Xenocelia* by Hieke (2001: 106).
Amara (Bradytus) coelestis Baliani, 1932: 16. Type material: 26 syntypes in BMNH, MCSNG and ZMHB, lectotype not yet designated. Type locality: China, Sichuan, Kangding (“Tatsienlu-Chiulung”). Transferred to subgenus *Celia* by Baliani (1937: 176); synonymized by Hieke (1975: 292).
Amara (Celia) expolita Baliani, 1934a: 191. Type material: Holotype male in BMNH, allotype in ZSIC, and 8 paratypes in BMNH, MCSNG and ZMHB, 18 more “syntypes” not yet accounted for. Type locality: India, “Punyab: Simla Hills, Baghi”. Synonymized by Hieke (1975: 299).

##### Diagnosis.

Adults of this species (Fig. 14) can be distinguished from those of all other species in the region by the following combination of character states: body length 7-8 mm; dorsal surface dark piceous; pronotum with both inner and outer basal impressions deeply impressed; elytra with parascutellar pore puncture absent; medial protibial spurs simple; tarsomere 5 of hind tarsi with two or (in a few specimens) three pairs of setae ventrally (Fig. 6b); last abdominal sternite of both male (Fig. 5d) and female (Fig. 5c) with two pairs of setiferous punctures near hind margin (second seta absent from one side in a few males for total of three setae).

##### Habitat distribution.

Specimens of this species were collected in daytime from under stones and other cover in open roadside areas (Fig. 23b) and other waste areas around human settlements (Fig. 23a) with scattered grasses and shrubs, at the edges of agricultural fields, and on open rocky, graveled and sandy banks of streams, and in these same habitats at night, when beetles were found active on bare substrate. They were especially conspicuous on open sandy beaches at night. Members of this species were found at elevations ranging from 1175 to 2800 m, syntopic with adults of *Amara birmana*, *Amara chalciope*, *Amara davidi*, *Amara dissimilis*, *Amara latithorax*, *Amara lucidissima*, *Amara shaanxiensis* and *Amara silvestrii* at one or more sites.

##### Geographical distribution within the Gaoligong Shan.

Fig. 14d. We examined a total of 164 specimens (92 males and 72 females) from the following localities: ***Fugong County*:** Lishadi Township (Shibali area, 27.16536°, 98.78003°, 2535 m, 6 October 2007, H.L. Shi collector [1 female; IOZ]), (0 to 2 km E of Shibali on Shibali Road, 27.16100°, 98.79370°, 2300–2350 m, 18 August 2005, D.Z. Dong collector [1 male and 1 female; IOZ]), (0.1 km below Shibali on Shibali Road, 27.16577°, 98.78091°, 2545 m, 5 May 2004, D.H. Kavanaugh & C.E. Griswold collectors [1 male; CAS]), (0.3 km SE of Shibali on North Fork Yamu He, 27.16337°, 98.78208°, 2475 m, 7 May 2004, D.H. Kavanaugh & C.E. Griswold collectors [1 male and 1 female; CAS]), (1.5 km below Shibali on Yaping Road, 27.16284°, 98.78989°, 2420 m, 2 May 2004, H.B. Liang & G.X. Peng collectors [1 male and 1 female; CAS, IOZ]), (Shilajia Village at Shibali Road, 27.13947°, 98.82184°, 1800–1900 m, 24–25 April 2004, D.H. Kavanaugh & C.E. Griswold collectors [2 females; CAS, IOZ]), (11 km above Nu Jiang on Yaping Road at Shimowa Village, 27.13839°, 98.82147°, 1850–1928 m, 25 April 2004, H.B. Liang collector [4 males and 5 females; CAS, IOZ]); Lumadeng Township (7.0 km SW of Lao Shibali on Lao Shibali Road at tributary of South Fork Yamu He, 27.10220°, 98.73107°, 2800 m, 13 August 2005, D.H. Kavanaugh & P. Paquin collectors [1 male; CAS]), (7.5 km below Shibali on Yaping Road, 27.14627°, 98.81559°, 2030 m, 3 May 2004, H.B. Liang & M. Xie collectors [2 males and 2 female; CAS, IOZ]), (South Fork Yamu He above Shilajia Village, 27.12101°, 98.83173°, 1630–1790 m, 26 April 2004, D.H. Kavanaugh collector [1 female; CAS]), (Yaping Road below Shibali, 27.16520°, 98.77980°, 2530 m, 24 April 2004, H.B. Liang & X.Y. Li collectors [1 male; IOZ]); Shangpa Township (Nu Jiang at west end of footbridge in Shangpa, 26.90743°, 98.86391°, 1175–1180 m, 20 April 2004, H.B. Liang collector [1 female; IOZ]). ***Gongshan County*:** Bingzhongluo Township (Bingzhongluo Village, 28.01940°, 98.62106°, 1760 m, 21 April 2002, H.B. Liang & W.D. Ba collectors [2 males and 1 female; IOZ, ZMHB]), (Gongdangshenshan, 27.99725°, 98.62003°, 2489 m, 12 November 2004, H.B. Liang collector [1 male; CAS]); Cikai Township (Heiwadi, 16.8 km W of Cikai on Dulong Valley Road, 27.79584°, 98.58443°, 2020 m, 20 April 2002, H.B. Liang & W.D. Ba collectors [2 males; IOZ]), (Heiwadi Village, 27.79584°, 98.58443°, 1965 m, 14 November 2004, H.B. Liang, D.Z. Dong & G. Tang collectors [26 males and 14 females; CAS, IOZ]), (North Fork Pula He above Heiwaidi Village, 27.78644°, 98.59831°, 1890 m, 15 November 2004, D.H. Kavanaugh & V.F. Lee collectors [3 males; CAS, IOZ]), (Nu Jiang in Cikai at Dashaba, 27.73845°, 98.67092°, 1430 m, 11 November 2003, D.H. Kavanaugh, H.B. Liang, D.Z. Dong & G. Tang collectors [1 female; CAS]), (Pula He just above Nu Jiang Road, 27.74861°, 98.66675°, 1440 m, 11 November 2004, D.H. Kavanaugh, H.B. Liang, D.Z. Dong & G. Tang collectors [1 female; CAS]); Dulongjiang Township (Bapo, Miliwang, 27.72383°, 98.36117°, 1956 m, 31 October 2004, H.B. Liang collector [21 males and 8 females; CAS, IOZ, ZMHB]), (0.6 km N of Dizhengdang Village on Dulong Jiang, 28.08442°, 98.32652°, 1880 m, 29–30 October 2004, D.H. Kavanaugh, D.Z. Dong & G. Tang collectors [4 males and 5 females; CAS, IOZ]), (S of Dizhengdang Village at Silalong He, 28.07654°, 98.32603°, 1890 m, 30 October 2004, D.H. Kavanaugh, D.Z. Dong & G. Tang collectors [2 males and 3 females; CAS, IOZ]), (Dizheng Wang, 28.08686°, 98.32840°, 1900–1970 m, 30 October 2004, D.H. Kavanaugh & D.Z. Dong collectors [1 female; CAS]), (Dulong Jiang at Elideng Village, 28.00287°, 98.32145°, 1640 m, 3 November 2004, D.H. Kavanaugh, H.B. Liang, D.Z. Dong & G. Tang collectors [1 male and 2 females; CAS, IOZ]), (Dulong Jiang at Xiajiudang Village, 27.94092°, 98.33340°, 1580 m, 4 November 2004, D.H. Kavanaugh, D.Z. Dong & G. Tang collectors [2 females; CAS, IOZ]), (0.5 km N of Kongdang, 27.88111°, 98.34063°, 1500 m, 25 October 2004, D.H. Kavanaugh, H.B. Liang, D.Z. Dong & G. Tang collectors [1 female; CAS], 5 November 2004, H.B. Liang collector [1 female; IOZ]), (2.3–3.3 airkm S of Longyuan Village on Dulong Jiang, 28.00532°, 98.32145°, 1685–1720 m, 2 November 2004, D.H. Kavanaugh, D.Z. Dong & G. Tang collectors [2 males and 1 female; CAS, IOZ]), (Maku, 27.68553°, 98.30425°, 1823 m, 2 November 2004, H.B. Liang collector [14 males and 13 females; CAS, IOZ]), (Siran Wang, 0.2 km above confluence with Dulong Jiang, 28.01347°, 98.32117°, 1720 m, 2 November 2004, D.H. Kavanaugh & D.Z. Dong collectors [1 female; CAS]). ***Lushui County*:** Luzhang Township (Yaojiaping He at Pianma Road, 25.97722°, 98.71091°, 2527 m, 20 May 2005, D.H. Kavanaugh, C.E. Griswold, H.B. liang, D.Z. Dong & G. Tang collectors [1 male; CAS]). ***Tengchong County*:** Jietou Township (Longtang He at Dahetou Lingganjiao, 25.73947°, 98.69630°, 2010 m, 20 May 2005, H.B. Liang & P. Hu collectors [1 female; IOZ]); Shangying Township (Km 42–46 on Bawan-Tengchong Road, 24.95361°, 98.74222°, 2290 m, 14 October 2003, H.B. Liang & X.C. Shi collectors [1 male; IOZ]).

Members of this species were collected from the northern to the southern parts of the study area (Core Areas 1, 2, 3, 5 and 6), but they were found only on the western side of the mountain range in the southern part (Core Area 6). This distribution pattern may be an artifact of inadequate sampling on the eastern slope of the mountain range in the south.

##### Overall geographical distribution.

Fig. 27. This species has been recorded from Bhutan, China (Gansu, Sichuan and Yunnan Provinces and Xizang Autonomous Region), India (Assam, Himachal Pradesh, Kashmir and Jammu, Sikkim and Uttar Pradesh), Nepal and Pakistan (northern). Its occurrence in the study area represents the southern limit of its known geographical range.

#### 7. Amara (Harpaloamara) latithorax

Baliani, 1934

http://species-id.net/wiki/Amara_latithorax

Figs 5b–c , 5e , 6b , 15 , 22b , 23b , 27 [Fig F26] [Fig F27] –30

Amara (Harpaloamara) latithorax Baliani, 1934a: 198. Type material: Holotype male, allotype, and 1 additional paratype in BMNH, 1 paratype in MCSNG, and 3 paratypes in FRSDD. Type locality: India, United Provinces, Kumaon, West Almora and Chakrata, Mundali.
Amara (Bradytus) interrupta Landin, 1955: 417. Type material: Holotype female in NHRS. Type locality: Myanmar (Burma), Kambaiti, 7000 ft. Synonymized by Hieke (1997: 227).
Amara (Bradytus) neglecta Landin, 1955: 416. Type material: Holotype male in NHRS. Type locality: Myanmar (Burma), Kambaiti, 2000 m. Synonymized by Hieke (1997:227).

##### Diagnosis.

**Figure 15.**
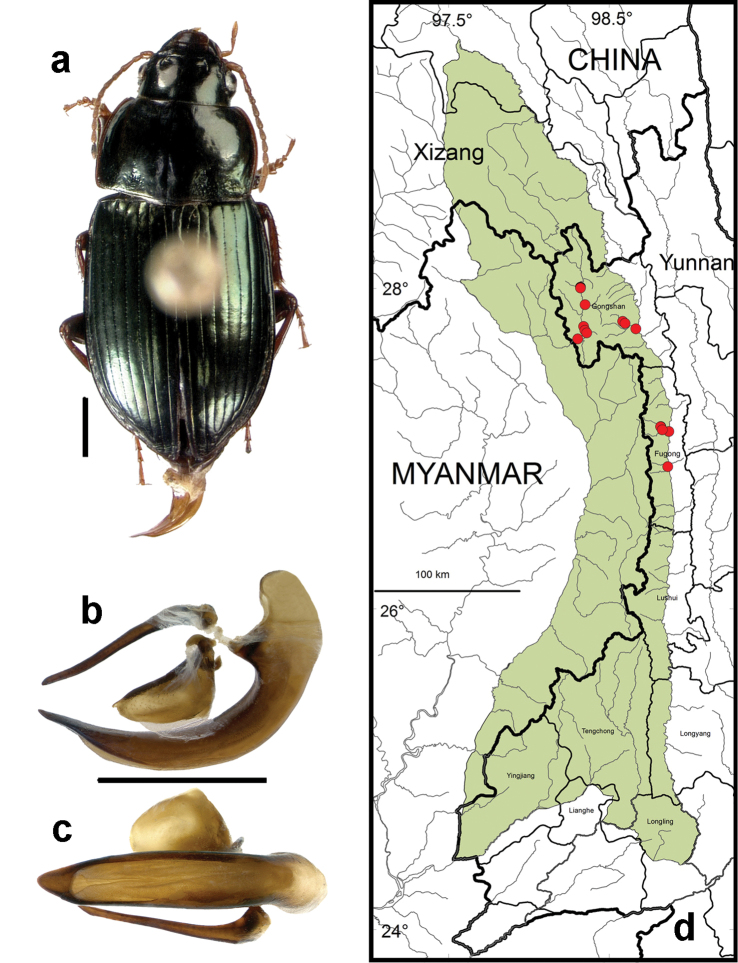
*Amara (Harpaloamara) latithorax* Baliani. **a** dorsal habitus (CASENT1015304); b-c. median lobe of aedeagus of male (CASENT10115091) **b** left lateral aspect **c** dorsal aspect; scale lines = 1.0 mm **d** Map of localities records (red circles) for *Amara* in the Gaoligong Shan region, scale line = 100 km.

Adults of this species can be distinguished from those of all other species in the region by the following combination of character states: body length 8.5-9.0 mm; dorsal surface black, with very faint metallic blue or green metallic reflection in most individuals, more vivid (as in Fig. 15a) in or lacking from a few specimens, at least femora and outer antennomeres dark (piceous to black); pronotum (Fig. 15a) only slightly narrowed anteriorly with anterior margin nearly straight and clearly narrower than posterior margin, without or with only very slightly projected anterior angles, posterior angles distinctly rounded (narrowly so in some individuals), slightly to moderately obtuse, pronotal base moderately coarsely punctate, outer basal impressions either not sharply delimited laterally by raised areas or, if so, then the raised area broader; elytra elytra with slight sub-basal depressions centered on striae 6 (visible on right elytron in Fig. 15a), also on striae 4 and/or 5 in some individuals, parascutellar pore puncture absent; medial protibial spurs simple; tarsomere 5 of hind tarsi with two or (in a few specimens) three pairs of setae ventrally (Fig. 6b); last abdominal sternite of male with one pair (Fig. 5e) and female with two pairs (Fig. 5c) of setiferous punctures near hind margin.

##### Habitat distribution.

Specimens of this species were collected in daytime from under stones and other cover in open roadside areas (Figs 22b, 23b) and meadows with scattered grasses and shrubs, at the edges of agricultural fields, including wet and dry rice paddies and on open sandy banks of streams, and in these same habitats at night, when beetles were found active on bare substrate. Members of this species were found at elevations ranging from 1195 to 2022 m, syntopic with adults of *Amara chalciope*, *Amara congrua*, *Amara dissimilis*, *Amara lucidissima* and *Amara sikkimensis* at one or more sites.

##### Geographical distribution within the Gaoligong Shan.

Fig. 15d. We examined a total of 37 specimens (12 males and 25females) from the following localities: ***Fugong County*:** Aludi Village (Nu Jiang, 27.10834°, 98.87218°, 1195-1250 m, 22 April 2004, D.H. Kavanaugh collector [1 female; CAS]); Lishadi Township (Shilajia Village at Shibali Road on North Fork Yamu He, 27.13947°, 98.82184°, 1800–1900 m, 24-25 April 2004, D.H. Kavanaugh & C.E. Griswold collectors [1 female; CAS]), (11 km above Nu Jiang on Yaping Road at Shimowa Village, 27.13839°, 98.82147°, 1850-1928 m, 25 April 2004, H.B. Liang collector [1 male and 2 females; CAS, IOZ, ZMHB]); Shangpa Township (road on west side of Nu Jiang S of Shangpa, 26.88952°, 98.86539°, 1223 m, 22 and 27 April 2004, D.H. Kavanaugh & C.E. Griswold collectors [1 male and 1 female; CAS, IOZ]). ***Gongshan County*:** Cikai Township (15 km W of Cikai on Dulong Valley Road, 27.79584°, 98.58443°, 2022 m, 10 October 2002, D.H. Kavanaugh, P.E. Marek & H.B. Liang collectors [1 female; CAS]), (Heiwadi Village, 27.72250°, 98.59902°, 1965 m, 14 November 2004, H.B. Liang, D.Z. Dong & G. Tang collectors [2 males; IOZ, ZMHB]), (Pula He just above Nu Jiang Road, 27.74861°, 98.66675°, 1440 m, 23 October 2004, D.H. Kavanaugh & H.B. Liang collectors [1 female; IOZ], 11 November 2004, D.H. Kavanaugh, H.B. Liang, D.Z. Dong & G. Tang collectors [1 male and 2 females; CAS, IOZ]); Dulongjiang Township (Bapo, 27.73902°, 98.34975°, 1412 m, 20 October 2004, H.B. Liang collector [1 female; IOZ], 3 November 2004, H.B. Liang collector [2 males and 1 female; CAS, IOZ], at Miliwang, 27.72383°, 98.36117°, 1956 m, 31 October 2004, H.B. Liang collector [6 females; CAS, IOZ]), (Dulong Jiang at Elideng Village, 28.00287°, 98.32145°, 1640 m, 3 November 2004, D.H. Kavanaugh, D.Z. Dong & G. Tang collectors [1 female; CAS]), (2.3-3.3 airkm S of Longyuan Village on Dulong Jiang, 28.00532°, 98.32145°, 1685-1720 m, 2 November 2004, D.H. Kavanaugh, D.Z. Dong & G. Tang collectors [1 female; IOZ], 2.8 km S of Longyuan Village on Dulong Jiang, 28.00905°, 98.32204°, 1660 m, 2 November 2004, D.H. Kavanaugh & D.Z. Dong collector [1 male; CAS]), (main road between Mabidang and Kongdang, 27.76361°, 98.34111°, 1329 m, 5 November 2004, V.F. Lee & D.G. Long collectors [1 female; CAS]), (Maku, 27.68553°, 98.30425°, 1823 m, 2 November 2004, H.B. Liang collector [2 males and 3 females; CAS, IOZ]), (Moqie Wang at KM 91 on Gongshan-Dulong Road, 27.89934°, 98.34999°, 1550 m, 6 November 2004, D.H. Kavanaugh & H.B. Liang collectors [1 male and 1 female; CAS, IOZ]).

Members of this species were collected only in the northern half of the study area (Core Areas 1, 2 and 3), on both sides of the mountain range (Core Areas 1 and 2).

##### Overall geographical distribution.

Fig. 27. This species has been recorded from Bhutan, China (Yunnan Province), India (Sikkim, Uttar Pradesh and West Bengal), Myanmar and Nepal. Its occurrence in the study area represents the southern and eastern limits of its known geographical range.

#### 8. Amara (Bradytus) chalciope

(Bates, 1891)

http://species-id.net/wiki/Amara_chalciope

Figs 5b–c , 5e , 6b , 16 , 24a , 24b , 25a , 26a , 27 [Fig F26] [Fig F27] –30

Leiocnemis chalciope Bates, 1891: 71. Type material: Holotype male in BMNH. Type locality: China, Sichuan, snowy mountain near Kangding (“Snowy Range, near Tatsienlu”), 13000 ft.
Amara (Niphobles) szetschuana Jedlička, 1934a: 17. Type material: Holotype female in NMPC. Type locality: China, Sichuan, Kangding (“Tatsienlu”). Transferred to subgenus *Bradytus* by Baliani (1937: 176), synonymized by Hieke (1983: 361).

##### Diagnosis.

**Figure 16.**
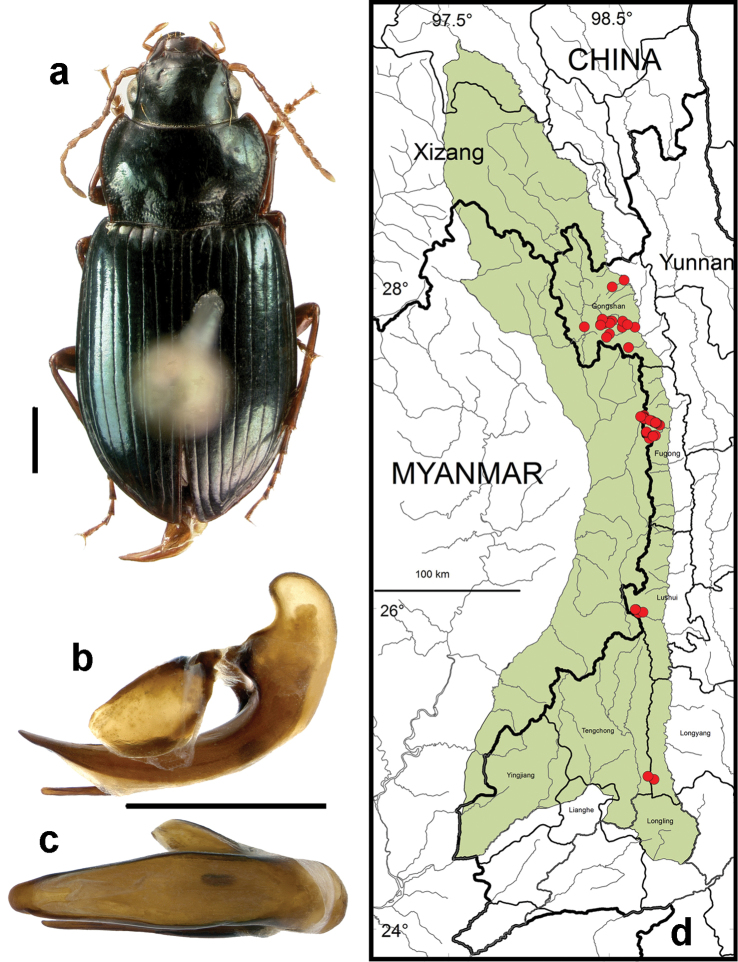
*Amara (Bradytus) chalciope* Bates. **a** dorsal habitus (CASENT1028265) **b–c** median lobe of aedeagus of male (CASENT1028375) **b** left lateral aspect **c** dorsal aspect; scale lines = 1.0 mm **d** Map of localities records (red circles) for *Amara chalciope* in the Gaoligong Shan region, scale line = 100 km.

Adults of this species (Fig. 16) can be distinguished from those of all other species in the region by the following combination of character states: body length 7.5–9.0 mm; dorsal surface dark with distinct metallic blue-green reflection in most specimens, non-metallic black in a few specimens, at least femora and outer antennomeres dark (piceous to black); elytral microsculpture comprised of isodiametric meshes, faintly impressed or nearly effaced in males, deeply impressed and distinct in females; pronotum (Fig. 16a) with lateral margins straight or faintly to distinctly sinuate just anterior to basal angles, rounded near middle, less rounded or nearly straight also in anterior one-third in most specimens, anterior angles distinctly and narrowly projected anteriorly beyond anterior margin, lateral explanation distinctly broader basally, outer basal impressions indistinct from lateral groove in most specimens, punctation of base extended anteriorly along sides to pronotal mid-length; elytra with parascutellar pore puncture absent; medial protibial spurs simple; metatibia of male without brush-like setae medially in the apical half; tarsomere 5 of hind tarsi with two or (in a few specimens) three pairs of setae ventrally (Fig. 6b); last abdominal sternite of male with one pair (Fig. 5e) and female with two pairs (Fig. 5c) of setiferous punctures near hind margin.

##### Habitat distribution.

Specimens of this species were collected in daytime from under stones and other cover in open roadside areas (Figs 24b, 25a, 26a), particularly on bare granitic sand substrates on roadcuts, landslides, stream banks, flood outwash flats and open slopes above treeline (Fig. 24a), and in these same habitats at night, when beetles were found active on bare substrate. Members of this species were found at elevations ranging from 1500 to 3611 m (most abundantly between 2500 and 3200 m), and syntopic with adults of *Amara birmana*, *Amara dissimilis*, *Amara elegantula*, *Amara latithorax*, *Amara lucidissima*, *Amara shaanxiensis*, *Amara sikkimensis*, *Amara silvestrii* at one or more sites.

##### Geographical distribution within the Gaoligong Shan.

Fig. 16d. We examined a total of 634 specimens (296 males and 338 females) from the following localities: ***Fugong County*:** Lishadi Township (Shibali area, 27.16553°, 98.77854°, 2550 m, 5 and7 May 2004, D.H. Kavanaugh, H.B. Liang, X.Y. Li, X.Q. Li and H.M. Yan collectors [12 females; IOZ]), 27.16536°, 98.78003°, 2535 m, 4–17 August 2005, D.H. Kavanaugh, H.B. Liang, P. Paquin & D.Z. Dong collectors [9 males and 7 females; CAS, IOZ], 4–5 October 2007, D.H. Kavanaugh, H.B. Liang & H.L. Shi collectors [1 male and 5 females]), (0.3 km SE of Shibali at North Fork Yamu River, 27.16337°, 98.78208°, 2475 m, 7 May 2004, D.H. Kavanaugh & C.E. Griswold collectors [2 males; CAS]),(1.5 km below Shibali on Yaping Road, 27.16284°, 98.78989°, 2420 m, 2 May 2004, H.B. Liang & G.X. Peng collectors [2 males and 6 females; CAS, IOZ]), (2.0 km E of Shibali on Shibali Road, 27.16276°, 98.78927°, 2430 m, 6 August 2005, H.B. Liang collector [5 males and 2 females; CAS, IOZ], 27.16100°, 98.79370°, 2300–2350 m, 18 August 2005, D.Z. Dong collector [3 males and 7 females; CAS, IOZ]), (2.7 km above of Shibali on Shibali Road, 27.17368°, 98.76684°, 2735 m, 10 August 2005, D.H. Kavanaugh collector [2 females; CAS, IOZ]), (2.8 km above of Shibali on Shibali Road, 27.17405°, 98.76722°, 2750 m, 9 August 2005, D.Z. Dong collector [1 male and 1 female; CAS, IOZ]), (4 km E of Shibali on Shibali Road, 27.15727°, 98.79784°, 2280 m, 11 August 2005, D.Z. Dong collector [1 male and 1 female; CAS IOZ]), (4 km W of Shibali on Shibali Road, 27.17740°, 98.75490°, 2800 m, 16 August 2005, D.Z. Dong collector [2 males and 1 female; CAS IOZ]), (8.4 to 9.5 km W of Shibali on Shibali Road, 27.18740°, 98.71936°, 3160–3195 m, 13 August 2005, D.H. Kavanaugh, H.B. Liang & D.Z. Dong collectors [1 male and 1 female; CAS, IOZ]), (8.5 km above Shibali on Shibali Road at North Fork Yamu River, 27.18416°, 98.72026°, 3100 m, 7 May 2004, D.H. Kavanaugh & B.X. Zhu collectors [1 male; CAS]), (9.5 to10.0 km W of Shibali on Shibali Road, 27.19438°, 98.71486°, 3195–3200m, 12 August 2005, D.H. Kavanaugh, H.B. Liang & D.Z. Dong collectors [15 males and 12 females; CAS, IOZ, ZMHB]), (10 km above Shibali on Shibali Road, 27.20055°, 98.71399°, 3121 m, 5–6 August 2005, D.H. Kavanaugh, P. Paquin & D.Z. Dong collectors [6 males and7 females; CAS, IOZ]), (10 km above Shibali on Shibali Road, 27.19980°, 98.71375°, 3200 m, 16 August 2005, J.F. Zhang collector [10 males and 9 females; CAS, IOZ]), (10 to 11 km above Shibali on Shibali Road, 27.19980°, 98.71375°, 3200–3280 m, 8 August 2005, D.H. Kavanaugh, H.B. Liang, P. Paquin & D.Z. Dong collectors [2 males and 3 females; CAS, IOZ]), (10.1 km above Shibali on Shibali Road, 27.20049°, 98.71354°, 3225 m, 6 May 2004, D.H. Kavanaugh, C.E. Griswold, H.B. Liang & B.X Zhu collectors [3 males and 1 female; CAS, IOZ]), (10.1 to11.5 km above Shibali on Shibali Road, 27.20676°, 98.71763°, 3225–3290 m, 8 May 2004, D.H. Kavanaugh, C.E. Griswold, H.B. Liang, X.Y. Li & Z.B. Xi collectors [1 male; IOZ]); Lumadeng Township (8.5 km above Shibali on Shibali Road at North Fork Yamu He, 27.18416°, 98.72026°, 3100 m, 8 August 2005, D.H. Kavanaugh, P. Paquin, D.Z. Dong & J.F. Zhang collectors [1 female; CAS], (7.5 km below Shibali on Yaping Road, 27.14627°, 98.81559°, 2030 m, 3 May 2004, H.B. Liang & M. Xie collectors [2 males; CAS, IOZ]), (Lao Shibali wood inspection station, 27.07831°, 98.77416°, 2305 m, 21 August 2005, H.B. Liang & J.F. Zhang collectors [2 males; CAS, IOZ]), (1 km E of Lao Shibali on Lao Shibali Road at South Fork Yamu He, 27.08141°, 98.78273°, 2275 m, 15 August 2005, D.H. Kavanaugh collector [1 males; CAS]), (1.3 km E of Lao Shibali on Lao Shibali Road at South Fork Yamu He, 27.08180°, 98.78670°, 2250 m, 15 August 2005, D.H. Kavanaugh, H.B. Liang & J.F. Zhang collectors [2 males and 6 females; CAS, IOZ]), (1.6 km E of Lao Shibali on Lao Shibali Road at South Fork Yamu He, 27.08260°, 98.78877°, 2240 m, 21 August 2005, D.H. Kavanaugh collector [5 males and 9 females; CAS, IOZ]), (6.7 km E of Lao Shibali on Lao Shibali Road at tributary of South Fork Yamu He, 27.10437°, 98.73253°, 2805 m, 13 August 2005, D.H. Kavanaugh, H.B. Liang, D.Z. Dong & J.F. Zhang collectors [1 male and 3 females; CAS, IOZ]), (7.0 km E of Lao Shibali on Lao Shibali Road at tributary of South Fork Yamu He, 27.10220°, 98.73107°, 2800 m, 13 August 2005, D.H. Kavanaugh & P. Paquin collectors [5 males; CAS, IOZ]); Lao Shibali Yakou (27.06429°, 98.75123°, 3270 m, 13 August 2005, D.H. Kavanaugh & D.Z. Dong collectors [1 male and 2 females; CAS, IOZ]); ridge and cirques S of Shibali Yakou (27.20035°, 98.69604°, 3599–3611 m, 5 October 2007, H.L. Shi, H.B. Liang & X.J. Peng collectors [1 female; IOZ]); South Fork Yamu He (at Lao Shibali, 27.07978°, 98.77328°, 2305 m, 15 August 2005, D.H. Kavanaugh & D.Z. Dong collectors [ 2 females; IOZ]), (at Yejiadi, 27.08004°, 98.77325°, 2307 m, 10 May 2004, H.B. Liang, X.Y. Li and B.X. Zhu collectors [3 males and 2 females; CAS, IOZ]). ***Gongshan County*:** Bapo (2 km N along Dulong Jiang, 27.76000°, 98.34611°, 1510 m, 16–17 July 2000, P. Thomas collector [2 males; CAS, IOZ]), Bingzhongluo Township (Guocai He at Fucai, 28.00855°, 98.51886°, 2800 m, 16 August 2006, D.Z. Dong collector [1 male; IOZ]), (Niwaluo He just above Nu Jiang Road, 28.05140°, 98.59319°, 1630 m, 8 October 2002, D.H. Kavanaugh, P.E. Marek & D.Z. Dong collectors [1 female; CAS]); Cikai Township (8.3 to 13.1 km NW of Cikai on Dulong Valley Road, 27.75653°, 98.58214°, 2630–3000 m, 23 September 2002, D.H. Kavanaugh, P.E. Marek & D.Z. Dong collectors [6 males and 10 females; CAS, IOZ]), (Km 49 on Gongshan-Dulong Road, 27.78075°, 98.47000°, 3330m, 1 October 2002, D.H. Kavanaugh collector [5 males and 6 females; CAS, IOZ]), (53 km W of Cikai on Dulong Valley Road, 27.77422°, 98.44716°, 3380 m, 1 October 2002, D.H. Kavanaugh & H.B. Liang collectors [2 females; CAS, IOZ]), (57 km W of Cikai on Dulong Valley Road, 27.80789°, 98.45736°, 3162 m, 2 October 2002, H.B. Liang collector [2 males and 1 female; CAS, IOZ]); Heipu Yakou ((southeast slope, 27.77032°, 98.44674°, 3365 m, 11and 13 August 2006, D.H. Kavanaugh, J.A. Miller, D.Z. Dong & Y. Liu collectors [6 males and 4 females; CAS, IOZ]); Dabadi (40 km W of Cikai on Dulong Valley Road, 27.79619°, 98.51867°, 3900 m, 29 September 2002, H.B. Liang collector [38 males and 28 females; CAS, IOZ, ZMHB]), (41 km W of Cikai on Dulong Valley Road, 27.79655°, 98.50562°, 3000 m, 27 September – 6 October 2002, D.H. Kavanaugh, P.E. Marek, H.B. Liang, D.Z. Dong & X.C. Li collectors [67 males and 70 females; CAS, IOZ, ZMHB]), (45 km W of Cikai on Dulong Valley Road,, 27.78253°, 98.50444°, 3133 m, 2 October 2002, H.B. Liang collector [2 males and 2 females; CAS, IOZ]); Danzhu He drainage (27.63063°, 98.62074°, 2700 m, 30 June-5 July 2000, D.H. Kavanaugh, C.E. Griswold & H.B. Liang collectors [20 males and 34 females; CAS, IOZ, ZMHB]); Heiwadi (15 km W of Cikai on Dulong Valley Road, 27.79584°, 98.58443°, 2022 m, 4 October 2002, H.B. Liang, W.D. Ba and C.G. Jin collectors [1 male and 1 female; CAS, IOZ], 10 October 2002, D.H. Kavanaugh, P.E. Marek & H.B. Liang collectors [11 males and 4 females; CAS, IOZ]), (27.77415°, 98.61382°, 1767 m, 5 November 2004, H.B. Liang collector [1 male; IOZ]); Qiqi He (27.75748°, 98.66073°, 1500 m, 30 September-1 October 2007, D.H. Kavanaugh, H.B. Liang & H.L. Shi collectors [1 female; CAS]); Qiqi Trail (at No. 12 Bridge Camp, 27.71502°, 98.50244°, 2775 m, 15–19 July 2000, D.H. Kavanaugh & H.B. Liang collectors [2 males and 1 female; CAS, IOZ]), (at Dongshaofang area, 27.69504°, 98.48433°, 3230–3680 m, 16–17 July 2000, D.H. Kavanaugh & H.B. Liang collectors [8 male and 10 females; CAS, IOZ]). ***Longyang County*:** Bawan-Tengchong Road Km 36–37 (24.93417°, 98.77944°, 2150 m, 12 October 2003, H.B.Liang & X.C. Shi collectors [1 female; IOZ]). ***Lushui County*:** Luzhang Township (Fengxue Yakou at Pianma Road, 25.97228°, 98.68336°, 3150 m, 15 October 2002, D.H. Kavanaugh P.E. Marek & H.B. Liang collectors [8 males and 10 females], 11 May 2005, D.H. Kavanaugh, H.B. Liang, C.E. Griswold, D.Z. Dong & G. Tang collectors [1 female; CAS]), (Yaojiaping He at Pianma Road, 25.97722°, 98.71091°, 2527 m, 19–20 May 2005, D.H. Kavanaugh, C.E. Griswold, H.B. Liang, D.Z. Dong & G. Tang collectors [23 males and 33 females; CAS, IOZ]); Pianma (9 km ESE on Pianma Road at Changya He, 25.99414°, 98.66336°, 2450 m, 14 May 2005, H.B. Liang collector [2 females; CAS, IOZ]), (9.3 km ESE on Pianma Road, 25.99363°, 98.66651°, 2460–2470m, 15–18 October 1998, D.H. Kavanaugh collector [6 males and 6 females; CAS, IOZ]), (Changya He, 25.99363°, 98.66651°, 2460–2470m, 13 October 1998, D.H. Kavanaugh collector [3 males and 6 females; CAS, IOZ]). ***Tengchong County*:** Bawan-Tengchong Road Km 42–46 (24.95361°, 98.74222°, 2290 m, 14 October 2003, H.B. Liang & X.C. Shi collectors [2 males and 1 female; CAS, IOZ].

This is the only species recorded from all seven Core Areas in the Gaoligong Shan region; but in the southern half of the study area, it is restricted to only the highest elevations, where members are found mainly on the passes over the crest of the mountain range and on the slopes just below the passes on both sides.

##### Overall geographical distribution.

Fig. 27. This species has been recorded from Bhutan and China (Fujian, Sichuan and Yunnan Provinces and Xizang Autonomous Region).

#### 9. Amara (Bradytus) dissimilis

Tschitschérine, 1894

http://species-id.net/wiki/Amara_dissimilis

Figs 5b–c , 5e , 6b , 17 , 22a , 24b , 27 [Fig F26] [Fig F27] –30

Amara (Bradytus) dissimilis Tschitschérine, 1894: 404. Type material: Holotype male in ZIN. Type locality: China, Gansu, Ponggartang (“Thibet sept., Amdo, village Ndàmi”). [Note: The holotype was erroneously labeled “*Brad. dissors* Tschit. 1894 typ!” by Hieke (1999a: 165).
Amara (Bradytus) emmerichi Baliani, 1932: 14. Type material: Holotype male (“type”) and one paratype in CBAL, additional paratypes in DEI (Döbler 1975: 112), NMPC and ZMHB. Type locality: China, Sichuan, Kangding (“Tatsienlu-Chiulung”). Synonymized by Hieke (1999a: 165).
Amara (Bradytus) lama Baliani, 1934b: 110. Type material: Holotype female and 1 paratype in BMNH, 2 paratypes in MCSNG. Type locality: Tibet, Rong Tö Valley, 4000-7000 ft. Synonymized by Hieke (1997: 225).
Amara (Bradytus) komala Jedlička, 1934b: 116. Type material: Holotype female in NMPC, 1 paratype female in CMEY. Type locality: China, Yunnan, Longchuan Jiang (“Soling-ho” Valley). Synonymized by Hieke 1995: 297.
Amara (Bradytus) mera Jedlička, 1934b: 116. Type material: Holotype female and 1 paratype female in NMPC. Type locality: China, Yunnan, “Yunnan-fou”. Synonymized by Hieke (1995: 297).

##### Diagnosis.

**Figure 17.**
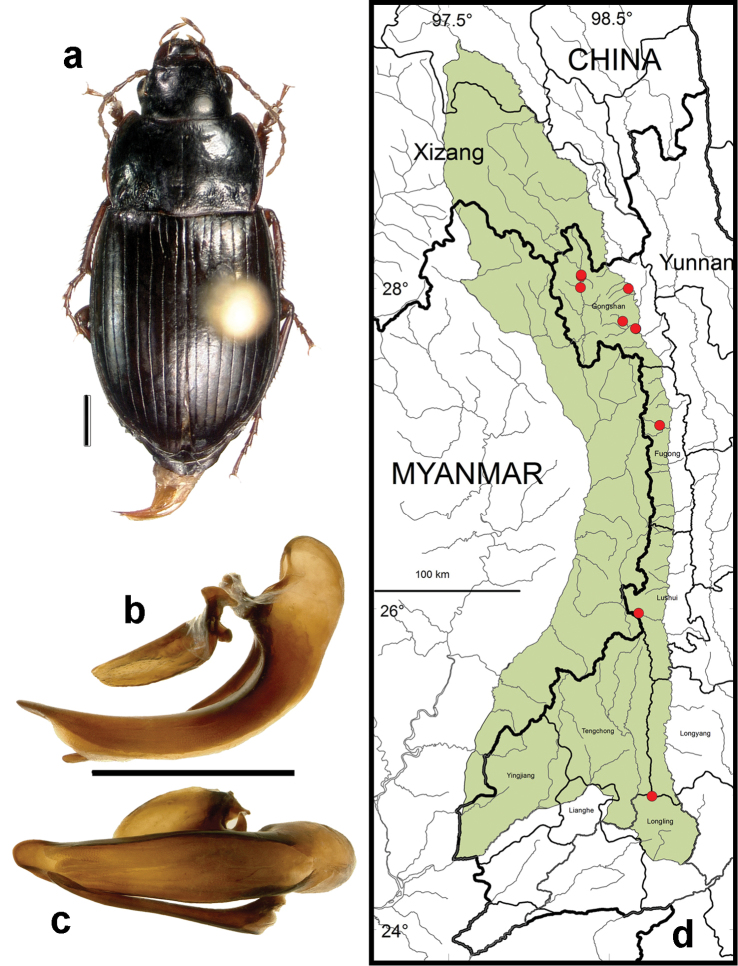
*Amara (Bradytus) dissimilis* Tschitschérine. **a** dorsal habitus (CASENT1033318) **b–c** median lobe of aedeagus of male (CASENT1002115) **b** left lateral aspect **c** dorsal aspect; scale lines = 1.0 mm **d** Map of localities records (red circles) for *Amara dissimilis* in the Gaoligong Shan region, scale line = 100 km.

Adults of this species (Fig. 17) can be distinguished from those of all other species in the region by the following combination of character states: body length 7.7–8.7 mm; dorsal surface dark brown to black, without or with only very faint metallic green reflection, at least femora and outer antennomeres dark (piceous to black); elytral microscuplture comprised of distinctly isodiametric meshes in both males and females (more deeply impressed in females than in males); pronotum (Fig. 17a) distinctly narrowed anteriorly, with anterior margin clearly narrower than the hind margin, lateral margins more or less evenly rounded from apical to basal angle, anterior angles slightly projected anteriorly beyond anterior margin, posterior angles obtusely angulate and slightly denticulate, basal impressions broadly and deeply foveate, outer basal impression distinctly delimited laterally by a broad convexity; elytra without evident sub-basal depressions, pore puncture absent, elytral striae distinct throughout and deeply impressed; medial protibial spurs simple; metatibiae of males with a brush-like patch of setae medially in the apical half; tarsomere 5 of hind tarsi with two or (in a few specimens) three pairs of setae ventrally (Fig. 6b); last abdominal sternite of male with one pair (Fig. 5e) and female with two pairs (Fig. 5c) of setiferous punctures near hind margin.

##### Habitat distribution.

Specimens of this species were collected in daytime from under stones and other cover in open roadside areas (Fig. 24b) and other waste areas (Fig. 22a) around human settlements with scattered grasses and shrubs, at the edges of meadows and agricultural fields, and on open rocky banks of streams, and in these same habitats at night, when beetles were found active on bare substrate. Members of this species were found at elevations ranging from 1500 to 3611 m (most abundantly between 1500 and 3150 m), and syntopic with adults of *Amara birmana*, *Amara chalciope*, *Amara latithorax*, *Amara lucidissima*, *Amara pingshiangi*, *Amara sikkimensis* and *Amara silvestrii* at one or more sites.

##### Geographical distribution within the Gaoligong Shan.

Fig. 17d. We examined a total of 14 specimens (7 males and 7 females) from the following localities: ***Fugong County*:** Lumadeng Township (7.5 km below Shibali on Yaping Road, 27.14627°, 98.81559°, 2030 m, 3 May 2004, H.B. Liang & M. Xie collectors [1 female; IOZ]). ***Gongshan County*:** Bingzhongluo Township (Gongdong, 27.99858°, 98.61933°, 2506 m, P.E. Marek collector [1 female; CAS]); Cikai Township (27.74939°, 98.66453°, 1515 m, 25 September 2002, H.B. Liang & W.D. Ba collectors [1 male and 1 female; IOZ]), (Pula He just above Nu Jiang Road, 27.74861°, 98.66675°, 1440m, 23 October 2004, D.H. Kavanaugh & H.B. Liang collectors [1 female; IOZ]); Dulongjiang Township (0.6 km N of Dizhengdang village on Dulong Jiang, 28.08442°, 98.32652°, 1880m, 29 October 2004, D.H. Kavanaugh, D.Z. Dong & G. Tang collectors [2 males; CAS, IOZ]), Dulongjiang Township (S of Dizhengdang village at Sialong He, 28.07654°, 98.32603°, 1890 m, 30 October 2004, D.H. Kavanaugh, D.Z. Dong & G. Tang collectors [1 female; CAS]), (2.3 to 3.3 airkm S of Longyuan village on Dulong Jiang, 28.00532°, 98.32145°, 1685–1720 m, 2 November 2004, D.H. Kavanaugh, D.Z. Dong & G. Tang collectors [2 females; CAS, IOZ]); Heiwadi (16.8 km W of Cikai on Dulong Valley Road, 27.79584°, 98.58443°, 2150 m, 4 October 2002, H.B. Liang, W.D. Ba & C.G. Jin collectors[1 male; IOZ]; 10 October 2004, D.H. Kavanaugh, P.E. Marek & H.B. Liang collectors [1 male; CAS]). ***Longyang County*:** Nankang Yakou (24.83167°, 98.76667°, 2130 m, 4 November 1998, D.H. Kavanaugh collector [1 males; CAS]). ***Lushui County*:** Luzhang Township (Fengxue Yakou at Pianma Road, 25.97228°, 98.68336°, 3150 m, 11 October 1998, D.H. Kavanaugh collector [1 male; CAS]).

This species was recorded from all but one Core Area (Core Area 5) in the Gaoligong Shan region. In the southern half of the study area, it is restricted to only the highest elevations, where members are found mainly on the passes over the crest of the mountain range.

##### Overall geographical distribution.

Fig. 27. This species has been recorded from Gansu, Qinghai, Shaanxi, Sichuan and Yunnan Provinces and Xizang Autonomous Region in China. Its occurrence in the study area represents the southern limit of its known geographical range.

#### 10. Amara (Bradytus) elegantula

Tschitschérine, 1899

http://species-id.net/wiki/Amara_elegantula

**Figure 18.**
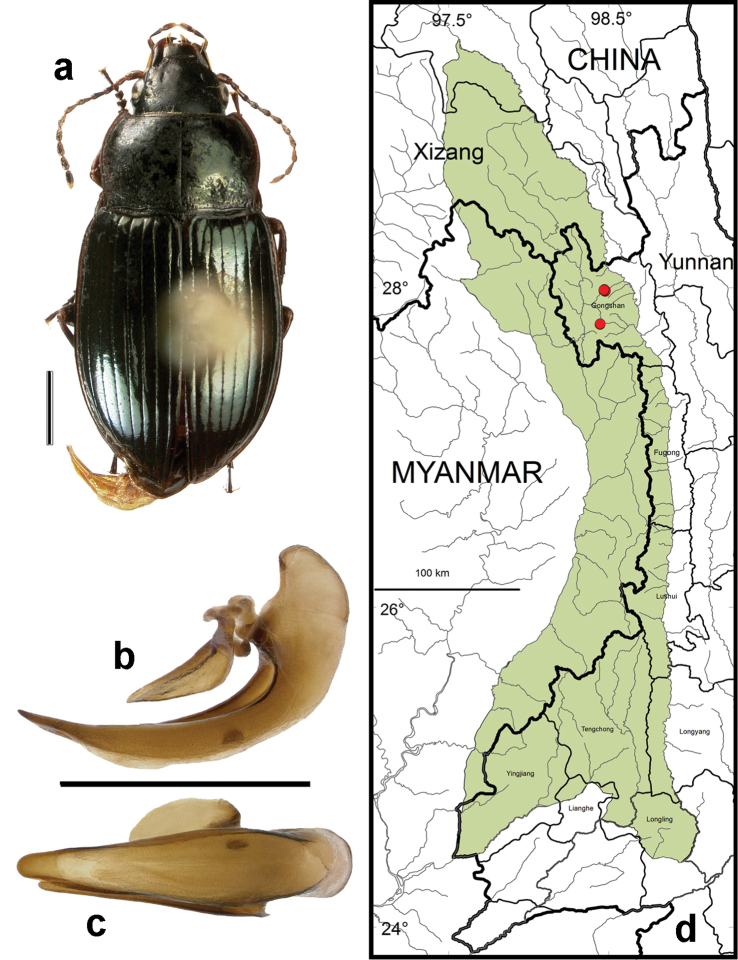
*Amara (Bradytus) elegantula* Tschitschérine. **a** dorsal habitus (CASENT1026671) **b–c** median lobe of aedeagus of male (CASENT1026255) **b** left lateral aspect **c** dorsal aspect; scale lines = 1.0 mm **d** Map of localities records (red circles) for *Amara elegantula* in the Gaoligong Shan region, scale line = 100 km.

Figs 5b–c , 5e , 6b , 18 , 26a , 26b , 27 [Fig F26] [Fig F27] –30

Amara (Leiocnemis) elegantula Tschitschérine, 1899: 659. Type material: a male (one of three syntypes) labeled “type” in ZIN acknowledged as holotype (Hieke 1981: 221). Type locality: Sikkim, Darjeeling, 12000 ft.

##### Diagnosis.

Adults of this species (Fig. 18) can be distinguished from those of all other species in the region by the following combination of character states: body length 7.0–7.5 mm; dorsal surface dark with distinct metallic blue-green reflection in most specimens, non-metallic black in a few specimens, at least femora and outer antennomeres dark (piceous to black); elytral microsculpture effaced or nearly so in both males and females; pronotum (Fig. 18a) with lateral margins straight or faintly to distinctly sinuate just anterior to basal angles, rounded near middle, less rounded or nearly straight also in anterior one-third in most specimens, anterior angles distinctly and narrowly projected anteriorly beyond anterior margin, lateral explanation narrow throughout, outer basal impressions foveate and distinct from lateral groove, punctation of base not extended anteriorly along sides beyond basal one-third; elytra with parascutellar pore puncture absent; medial protibial spurs simple; tarsomere 5 of hind tarsi with two or (in a few specimens) three pairs of setae ventrally (Fig. 6b); last abdominal sternite of male with one pair (Fig. 5e) and female with two pairs (Fig. 5c) of setiferous punctures near hind margin; male aedeagus with apical third of median lobe about as broad as middle third, apical hook of right paramere smaller and closer to apex (Fig. 8a).

##### Habitat distribution.

Specimens of this species were collected in daytime from under stones and other cover in open roadside areas near the crest of the mountain range (Fig. 26a), on the rocky heath tundra in areas with scattered vegetation, and on stabilized scree slopes between persistent snow patches in glacial cirques (Fig. 26b). At night, specimens were found in these same areas active on the bare substrate. Specimens were found at elevations ranging from 3350 to 4000 m, the highest sites sampled. The only other *Amara* species members of which were found syntopic with those of *Amara elegantula* was *Amara chalciope*, with adults of both species found together at only one site at an elevation of 3365 m (Fig. 26a), near the lower altitudinal limit of the former and the upper altitudinal limit of the latter.

##### Geographical distribution within the Gaoligong Shan.

Fig. 18d. We examined a total of 60 specimens (30 males and 30 females) from the following localities: ***Gongshan County*:** Heipu Yakou (northwest slope along road W of tunnel, 27.77447°, 98.44793°, 3350 m, 13 August 2006, D.H. Kavanaugh & J.A. Miller collectors [2 females; CAS, IOZ]), (southeast slope, 27.77032°, 98.44674°, 3365 m, 11–13 August 2006, D.H. Kavanaugh, J.A. Miller, D.Z. Dong & Y. Liu collectors [15 males and 9 females; CAS, IOZ]); southwest slope of Kawakarpu Shan (on slope NE of Chukuai Lake, 27.98206°, 98.48027°, 3950 m, 20 August 2006, Y. Liu, P. Hu, D.Z. Dong & J. Wang collectors [4 males and 3 females; CAS, IOZ]), (0.9 km N of Chukuai Lake, 27.98981°, 98.47392°, 4000 m, 21 August 2006, D.H. Kavanaugh, J. Xiong & C.H. Li collectors [2 females; CAS, IOZ]), (0.3 km NNE of Chukuai Lake, 27.98393°, 98.47491°, 3745 m, 19 August 2006, D.H. Kavanaugh, J.A. Miller, D.Z. Dong, J. Xiong & C.H. Li collectors [7 males and 8 females; CAS, IOZ, ZMHB]), (0.4 km NW of Chukuai Lake, 27.98231°, 98.47069°, 3808 m, 21 October 2006. D.Z. Dong collector [2 males; CAS, IOZ]), (0.75 km NW of Chukuai Lake, 27.98631°, 98.47069°, 3820 m, 21 August 2006, Y. Liu, P. Hu & J. Wang collectors [1 male and 2 females; CAS, IOZ]), (0.3 km SW of Chukuai Lake, 27.97686°, 98.47799°, 3750 m, 19 August 2006, Y. Liu collector [2 males and 3 females; CAS, IOZ]).

This species was recorded only from the northern part of the study area (Core Areas 1 and 2), where it is restricted to the highest elevations sampled, along the crest of the mountain range and both east and west slopes just below the crest.

##### Overall geographical distribution.

Fig. 27. This species has been recorded from Bhutan, China (Yunnan Province and Xizang Autonomous Region), India (Sikkim and West Bengal) and eastern Nepal. Its occurrence in the study area represents the southern and eastern limits of its known geographical range.

#### 11. Amara (Bradytus) simplicidens

Morawitz, 1863

http://species-id.net/wiki/Amara_simplicidens

**Figure 19.**
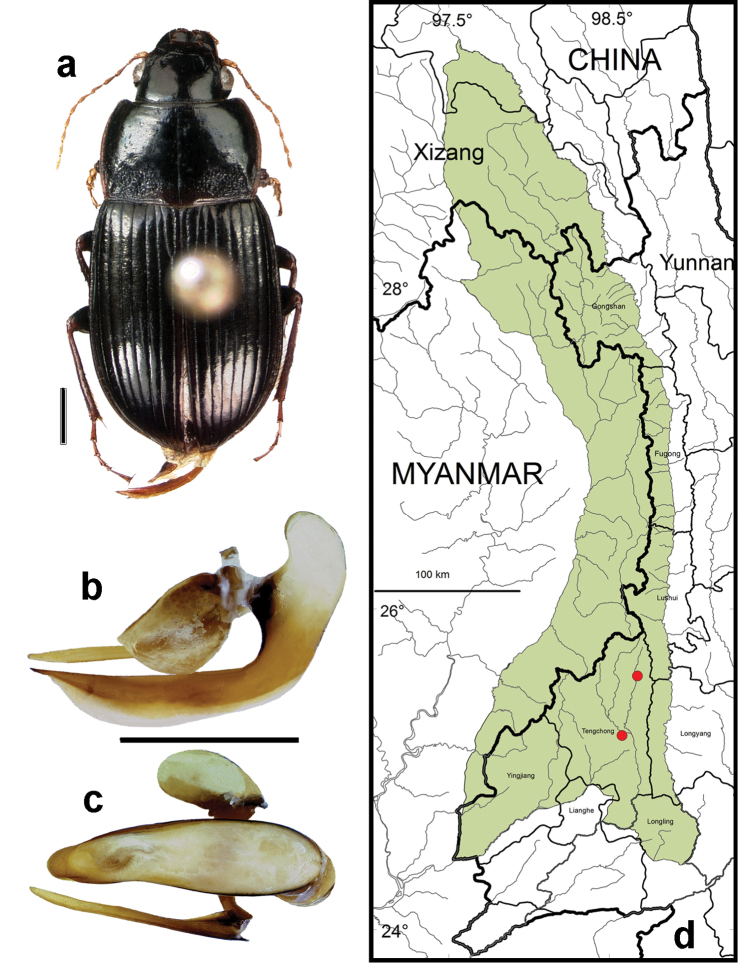
*Amara (Bradytus) simplicidens* Morawitz **a** dorsal habitus (CASENT1013861) **b–c** median lobe of aedeagus of male (CASENT1013861) **b** left lateral aspect **c** dorsal aspect; scale lines = 1.0 mm **d** Map of localities records (red circles) for *Amara simplicidens* in the Gaoligong Shan region, scale line = 100 km.

Figs 5b–c , 5e , 6b , 19 , 27 [Fig F26] [Fig F27] –30

Amara (Bradytus) simplicidens Morawitz, 1863b: 60. Type material: 3 syntypes (2 males and 1 female) in ZIN (lectotype not yet designated). Type locality: Japan, Hokkaido, Hakodate.
Amara (Bradytus) punctatissima Baliani, 1932: 13. Type material: Holotype male, allotype and at least 1 other paratype in MCSNG, 1 paratype in DEI. Type locality: China, Sichuan, Kangding (“Tatsienlu, Grenze O.Tibet”). Synonymized by Hieke (1995: 301).
Amara (Leiocnemis) marginicollis , Lutshnik, 1915: 130. Type material: Holotype female, originally in Lutshnik collection, but now missing (Hieke 1999a: 171). Type locality: Japan, Harima. Synonymized by Hieke (1999a: 171).
Amara (Leiocnemis) matsumurae Csiki, 1929: 450, new name for *Amara marginicollis* Lutshnik, 1915, [nec Morawitz 1863a:259]. Synonymized by Hieke (1999a: 171).

##### Diagnosis.

Adults of this species (Fig. 19) can be distinguished from those of all other species in the region by the following combination of character states: body length 8.3–8.4 mm; dorsal surface dark brown to black, without metallic reflection, at least femora and outer antennomeres dark (piceous to black); elytral microscuplture comprised of distinctly transverse meshes in both males and females (more transverse and less deeply impressed in males than in females); pronotum (Fig. 19a) only slightly narrower anteriorly than basally, anterior margin almost as wide as posterior margin, lateral margins more or less evenly rounded from apical to basal angle, anterior angles slightly projected anteriorly beyond anterior margin, posterior angles sharp, not rounded, outer basal impressions sharply delimited laterally by narrow, slightly oblique raised (but not carinate) areas, pronotal base very coarsely punctate; elytra with parascutellar pore puncture absent; medial protibial spurs simple; metatibia of male with brush-like setae medially in the apical half; tarsomere 5 of hind tarsi with two or (in a few specimens) three pairs of setae ventrally (Fig. 6b); last abdominal sternite of male with one pair (Fig. 5e) and female with two pairs (Fig. 5c) of setiferous punctures near hind margin.

##### Habitat distribution.

Specimens of this species were collected in daytime from under stones on the open bank of a small stream at an elevation of 1740 m and at night on wet stones along a roadside at 1515 m elevation. The only other *Amara* species members of which were found syntopic with those of *Amara simplicidens* was *Amara lucidissima*, with adults of both species found together at the 1740 m site.

##### Geographical distribution within the Gaoligong Shan.

Fig. 19d. We examined a total of 2 specimens (1 male and 1 female) from the following localities: ***Tengchong County*:** Datang Village (Maluchong, 25.58194°, 98.67583°, 1740 m, 24 October 2003, H.B. Liang collector [1 male; CAS]); Longchuan Jiang (at Xiangyang Bridge, 25.21056°, 98.58028°, 1515 m, 23 October 2003, H.B. Liang & X.C. Shi collectors [1 female; IOZ]).

This species was recorded only from the western slope of the southern part of the study area (Core Area 6).

##### Overall geographical distribution.

Fig. 27. This species has been recorded from China (Fujian, Heilongjiang, Henan, Hubei, Jiangsu, Jiangxi, Sichuan,Yunnan, and Zhejian Provinces), Japan, North Korea, and Russia (Khabarovsk and Primorsky Kraya, Sakhalinskaya Oblast, and the Kuril Islands). Its occurrence in the study area represents the western limit of its known geographical range.

#### 12. Amara (Bradytus) pingshiangi

Jedlička, 1957

http://species-id.net/wiki/Amara_pingshiangi

**Figure 20.**
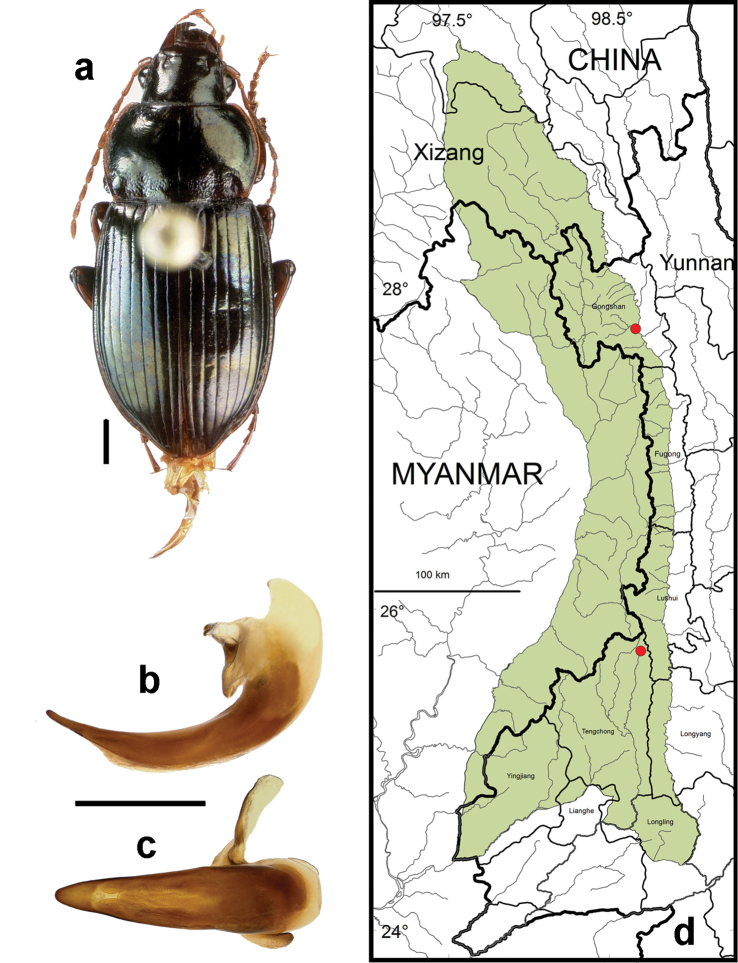
*Amara (Bradytus) pingshiangi* Jedlička. **a** dorsal habitus (CASENT1038324) **b–c** median lobe of aedeagus of male (CASENT1038324) **b** left lateral aspect **c** dorsal aspect; scale lines = 1.0 mm **d** Map of localities records (red circles) for *Amara pingshiangi* in the Gaoligong Shan region, scale line = 100 km.

Figs 5b–c , 5e , 6a , 20 , 25b , 27 [Fig F26] [Fig F27] –30

Amara (Curtonotus) pingshiangi Jedlička, 1957: 24. Type material: Lectotype female in NMPC (Hieke 1990: 238). Type locality: China, “Süd China: Pingshiang”, probably Jiangsu Province. Transferred to subgenus *Bradytus* by Hieke (1990: 238).

##### Diagnosis.

Adults of this species (Fig. 20) can be distinguished from those of all other species in the region by the following combination of character states: body length 11–13 mm, body form stout; dorsal surface dark piceous, at least femora and outer antennomeres dark (piceous to black); pronotum with lateral margin markedly rounded,pronotal base punctate, lateral areas in basal half and near anterior margin also punctuate in most specimens; elytra with parascutellar pore puncture absent; medial protibial spurs simple; tarsomere 5 of hind tarsi with five or six pairs of setae ventrally (Fig. 6a); last abdominal sternite of male with one pair (Fig. 5e) and female with two pairs (Fig. 5c) of setiferous punctures near hind margin.

##### Habitat distribution.

Specimens of this species were collected only at night, active on the ground in marshy meadow margin areas (Fig. 25b) near forest edges, at elevations ranging from 1515 to 2010 m, and syntopic with adults of *Amara birmana*, *Amara dissimilis* and *Amara shaanxiensis* at one or more sites.

##### Geographical distribution within the Gaoligong Shan.

Fig. 20d. We examined a total of 3 specimens (2 males and 1 female) from the following localities: ***Gongshan County*:** Cikai Township (27.74939°, 98.66453°, 1515 m, 25 Sep. 2002, H.B. Liang & W.D. Ba collectors [1 female; IOZ]). ***Tengchong County*:** Longtang He (at Dahetou Lingganjiao, 25.73947°, 98.6963°, 2010 m, 16 May 2006, D.H. Kavanaugh collector [1 male; CAS], 18 May 2006, D.H. Kavanaugh & R.L. Brett collectors [1 male; CAS]).

Members of this species were collected at only two sites, one in the northeastern part (Core Area 2) and other in the southwestern part (Core Area 6) of the study area, but not in intervening areas. This gap in distribution is most likely an artifact of inadequate sampling and not a real disjunction.

##### Overall geographical distribution.

Fig. 27. This species has been recorded from Fujian, Jiangxi, Sichuan, Yunnan and Zhejiang Provinces in China. Its occurrence in the study area represents the western limit of its known geographical range.

#### 13. Amara (Reductocelia) lucidissima

Baliani, 1932

http://species-id.net/wiki/Amara_lucidissima

Figs 5b–c , 5e , 6b , 8a , 21 , 22a , 27 [Fig F26] [Fig F27] –30

Amara (Celia) lucidissima Baliani, 1932: 10. Type material: Holotype male and allotype in MCSNG, 2 female paratypes in NMPC, and 1 female paratype in ZMHB. Type locality: China, Sichuan, Kangding (“Tatsienlu-Chiulung”). Synonymized with *Amara alacris* Tschitschérine by Hieke (1975: 316), again treated as a distinct species by Hieke (1994: 320), and transferred to subgenus *Reductocelia* by Hieke (1999b: 350).
Amara (Leiocnemis) kuatensis Jedlička, 1956: 209. Type material: Holotype male in ZFMK, about 200 paratypes in NMPC and ZFMK. Type locality: China, Fujian, Kuatun. Synonymized with *Amara alacris* Tschitschérine by Hieke (1975: 312), later synonymized with *Amara lucidissima* by Hieke (1994: 320).

##### Diagnosis.

**Figure 21.**
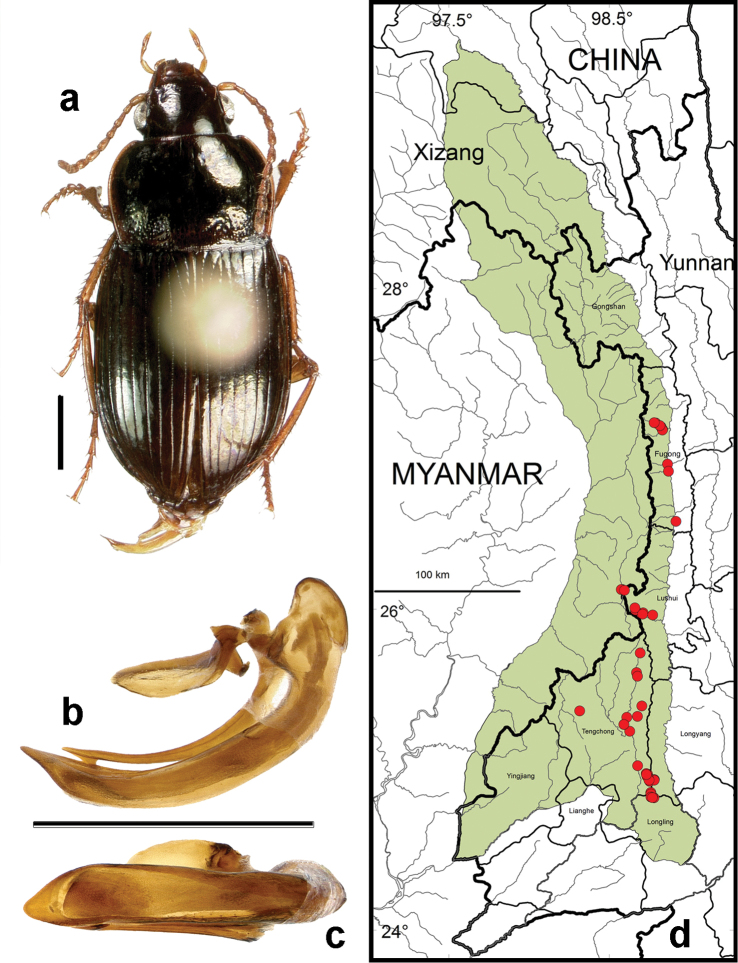
*Amara (Reductocelia) lucidissima* Baliani. **a** dorsal habitus (CASENT1011863) **b–c** median lobe of aedeagus of male (CASENT1002221) **b** left lateral aspect **c** dorsal aspect; scale lines = 1.0 mm **d** Map of localities records (red circles) for *Amara lucidissima* in the Gaoligong Shan region, scale line = 100 km.

Adults of this species (Fig. 21) can be distinguished from those of all other species in the region by the following combination of character states: body length 6.5–8.0 mm; dorsal surface light-brown to brownish black, without metallic reflection, entire legs and antennae pale; elytra with parascutellar pore puncture absent; medial protibial spurs simple; tarsomere 5 of hind tarsi with two or (in a few specimens) three pairs of setae ventrally (Fig. 6b); last abdominal sternite of male with one pair (Fig. 5e) and female with two pairs (Fig. 5c) of setiferous punctures near hind margin; male aedeagus with apical third of median lobe broader than middle third, apical hook of right paramere large and slightly subapical (Fig. 8a).

##### Habitat distribution.

**Figure 22.**
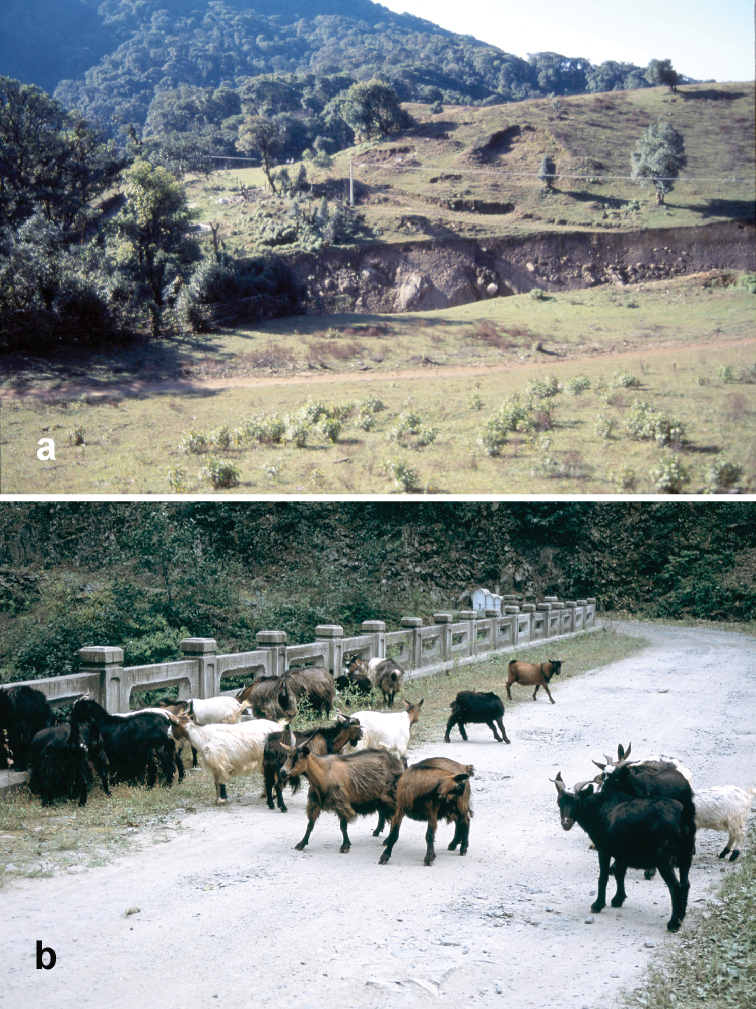
Photographs of habitats for *Amara* species in the Gaoligong Shan region. a. Baoshan-Tengchong Road at Nankang Yakou, elevation 2130 m; habitat in which specimens of *Amara birmana*, *Amara davidi*, *Amara dissimilis*, *Amara lucidissima* and *Amara shaanxiensis* were collected. b. Gongshan County, Cikai Township, Heiwadi Village area, elevation 1800 m; habitat in which specimens of *Amara congrua* and *Amara latithorax* were collected.

Specimens of this species were collected, often in great abundance, in daytime from under stones and other cover in open roadside areas and waste areas (Fig. 22a) around human settlements with scattered grasses and shrubs, at the edges of agricultural fields and on open banks of streams, and in these same habitats at night, when beetles were found active on bare substrate. Members of this species were found at elevations ranging from 1185 to 3140 m and syntopic with adults of *Amara birmana*, *Amara chalciope*, *Amara dissimilis*, *Amara latithorax*, *Amara shaanxiensis*, *Amara sikkimensis*, *Amara simplicidens* and *Amara silvestrii* at one or more sites.

##### Geographical distribution within the Gaoligong Shan.

Fig. 21d. We examined a total of 581 specimens (276 males and 305 females) from the following localities: ***Fugong County*:** Mugujia Village (Guquan He, 26.86177°, 98.86900°, 1175 m, 21 April 2004, D.H. Kavanaugh collector [1 female; CAS]); Shibali Road (above Shilajia village, 27.13947°, 98.82184°, 1900 m, 24 April 2004, D.H. Kavanaugh collector [2 females; CAS]), (above Shilajia Village at North Fork Yamu He, 27.13947°, 98.82184°, 1800–1900 m, 25 April 2004, D.H. Kavanaugh & C.E. Griswold collectors [18 males and 8 females; CAS, IOZ]); South Fork Yamu He (above Shilajia Village, 27.12101°, 98.83173°, 1630–1790 m, D.H. Kavanaugh collector [1 male; CAS]). ***Longyang County*:** Bawan-Tengchong Road Km 36 (24.9375°, 98.78028°, 2075 m, 11 October 2003, H.B.Liang collector [2 males and 2 females; CAS, IOZ]); Bawan-Tengchong Road Km 48–51 near Dahaoping Forest Station (24.97556°, 98.73000°, 2014 m, 18 October 2003, H.B. Liang collector [1 male, 3 females; CAS, IOZ]); Bawan-Tengchong Road Km 40–41 (24.92694°, 98.75278°, 2404 m, 12 October 2003, H.B.Liang collector [7 males and 3 females; CAS, IOZ]); Bawan-Tengchong Road Km 51 near Dahaoping Forest Station (24.9725°, 98.73889°, 2170 m, 18 October 2003, H.B. Liang & X.C. Shi collectors [1 female; IOZ]); Nankang Forest Station (24.82444°, 98.77889°, 2085 m, 27 October 2003, H.B. Liang & X.C. Shi collectors [12 males, 6 females; CAS, IOZ]); Nankang Yakou (24.83167°, 98.76667°, 2130 m, 4–7 November 1998, D.H. Kavanaugh collector [23 males and 44 females; CAS, IOZ]), (at Baoshan-Tengchong Road Km 24, 24.82583°, 98.77222°, 2130 m, 26 October 2003m, H.B. Liang & X.C. Shi collectors [11 males and 10 females; CAS, IOZ]), (mountain NW of Nankang Yakou, 24.83250°, 98.76944°, 2245 m, 27 October 2003, H.B. Liang & X.C. Shi collectors [1 male and 1 female; CAS, IOZ]). ***Lushui County*:** Gangfang Sancha Lukou (26.12167°, 98.575°, 1550 m, 14–15 October 1998, D.H. Kavanaugh collector [3 males and 5 females; CAS, IOZ]). ***Tengchong County*:** Bawan-Tengchong Road Km 42–46 (24.95361°, 98.74222°, 2290 m, 14 October 2003, H.B. Liang & X.C. Shi collectors [2 males and 1 female; IOZ], 17 October 2003, H.B. Liang collector [1 male and 2 females; IOZ]); Bawan-Tengchong Road Km 46–51 (24.95722°, 98.73667°, 2220 m, 17 October 2003, D.Z. Dong, H.B. Liang & X.C. Shi collectors [16 males and 25 females; CAS, IOZ]); Bawan-Tengchong Road Km 65 at Longwenqiao (25.02396°, 98.67675°, 1285 m, 19 October 2003, D.Z. Dong collector [4 males and 1 female; CAS, IOZ]); Baoshan-Tengchong Road Km 24 (24.82889°, 98.76028°, 2008 m, 29 October 2003, N.D. Penny & T.S. Briggs collectors [5 males and 6 females; CAS, IOZ]); 5–8 km E of Dahaoping (24.93417°, 98.7475°, 2358 m, 18 October 2003, N.D. Penny, T.S. Briggs, & D.Z. Dong collectors [1 male; CAS]); Datang Village (Maluchong, 25.58194°, 98.67583°, 1740 m, 24 October 2003, H.B. Liang collector [1 male; IOZ]), (Danlonghe Bridge, 25.60500°, 98.67028°, 1768 m, 24October 2003, H.B. Liang & X.C. Shi collectors [3 males and 3 females; CAS, IOZ]); Longchuan Jiang (at Longkou Village, 25.28167°, 98.59167°, 1500 m, 2 November 1998, D.H. Kavanaugh & C.E. Griswold collectors [4 males and 11 females; CAS, IOZ]), (at Xiaojiangqiao (25.23944°, 98.62722°, 1445 m, 21 October 2003, H.B. Liang & X.C. Shi collectors [1 male and 2 females; CAS, IOZ]), (at Yonganqiao (25.32556°, 98.60944°, 1500 m, 22 October 2003, H.B. Liang & X.C. Shi collectors [10 male and 10 females; CAS, IOZ]), (at Yonganqiao (25.60500°, 98.67583°, 1500 m, 22 October 2003, H.B. Liang & X.C. Shi collectors [2 females; IOZ]); Longkou Village, 25.28167°, 98.59167°, 1500 m, 22 October 2003, H.B. Liang & X.C. Shi collectors [15 males and 26 females; CAS, IOZ]); Shaba Village (25.39639°, 98.70306°, 1850 m, 23 October 2003, H.B. Liang & X.C. Shi collectors [6 males and 7 female; CAS, IOZ]); Xiaodifang Village (24.85722°, 98.75917°, 2150 m, 29 October 2003, D.Z. Dong collector [2 males and 1 female; CAS, IOZ]); Xiaoheishan Forest Station (24.82889°, 98.76000°, 2025 m, 29 October 2003, H.B. Liang collector [32 males and 41 females; CAS, IOZ]); Zhoujia-po Village, 25.33222°, 98.67611°, 1740 m, 24 October 2003, D.Z. Dong collector [2 males; CAS]); near Zhoujia-po village (25.58194°, 98.67611°, 1725 m, 24 October 2003, N.D. Penny & T.S. Briggs collectors [2 females; CAS, IOZ].

This species was recorded from the southern two-thirds of the study area (Core Areas 2,4, 5, 6 and 7), on both eastern and western slopes of the mountain range.

##### Overall geographical distribution.

Fig. 27. This species has been recorded from China (Fujian, Hubei, Sichuan, Yunnan, and Zhejiang Provinces) and Taiwan. Its occurrence in the study area represents the western limit of its known geographical range.

**Figure 23.**
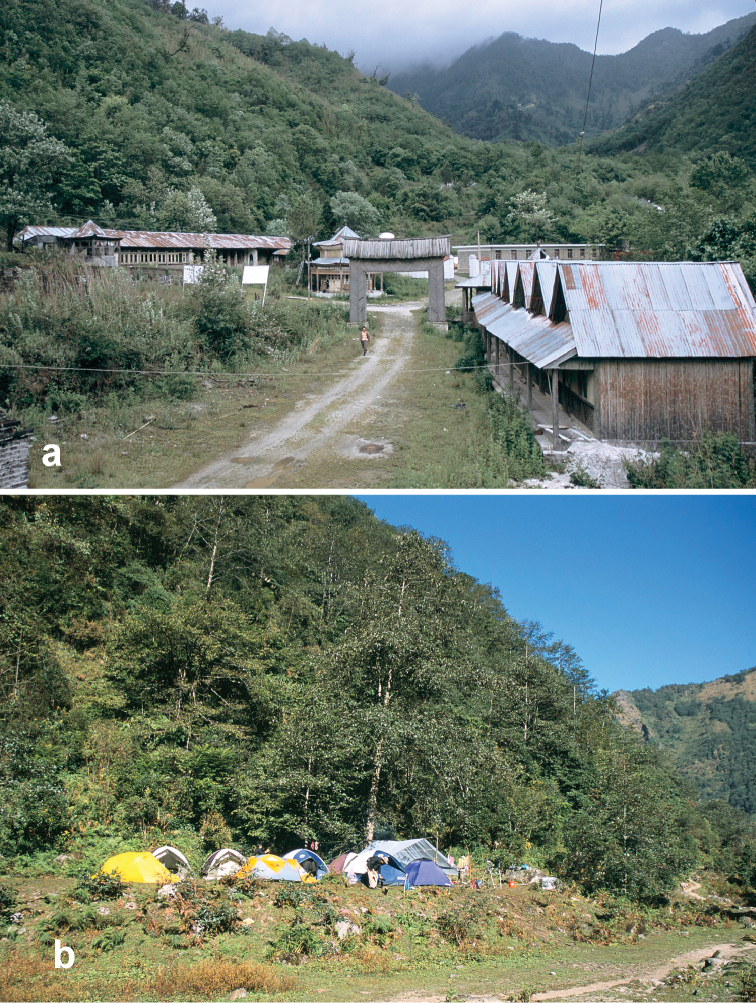
Photographs of habitats for *Amara* species in the Gaoligong Shan region. **a** Lushui County, Yaojiaping just off Pianma Road, elevation 2600 m; habitat in which specimens of *Amara birmana*, *Amara sikkimensis* and *Amara silvestrii* were collected **b** Gongshan County, Dulong Valley, 2.8 km S of Longyuan Village, elevation 1660 m; habitat in which specimens of *Amara latithorax* and *Amara sikkimensis* were collected.

**Figure 24.**
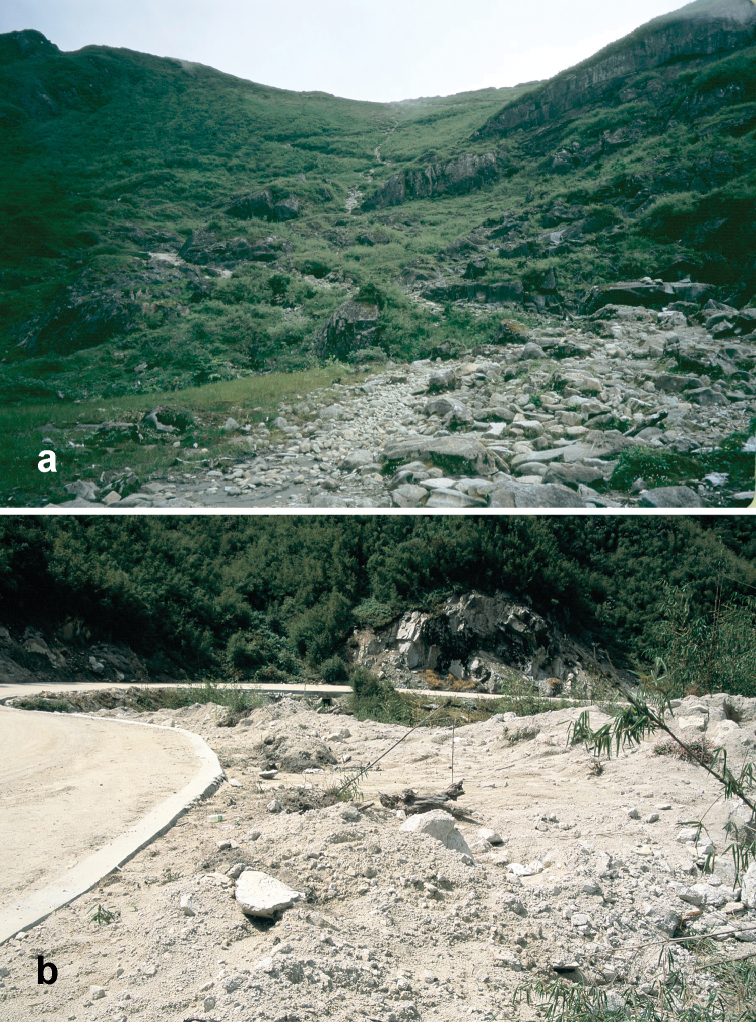
Photographs of habitats for *Amara* species in the Gaoligong Shan region. **a** Gongshan County, Dongshaofang area just below pass into Dulong Valley, elevation 3500 m; habitat in which specimens of *Amara chalciope* were collected **b** Lushui County, Pianma Road just W of Fengxue Yakou, elevation 3150 m; habitat in which specimens of *Amara dissimilis* and *Amara chalciope* were collected.

**Figure 25.**
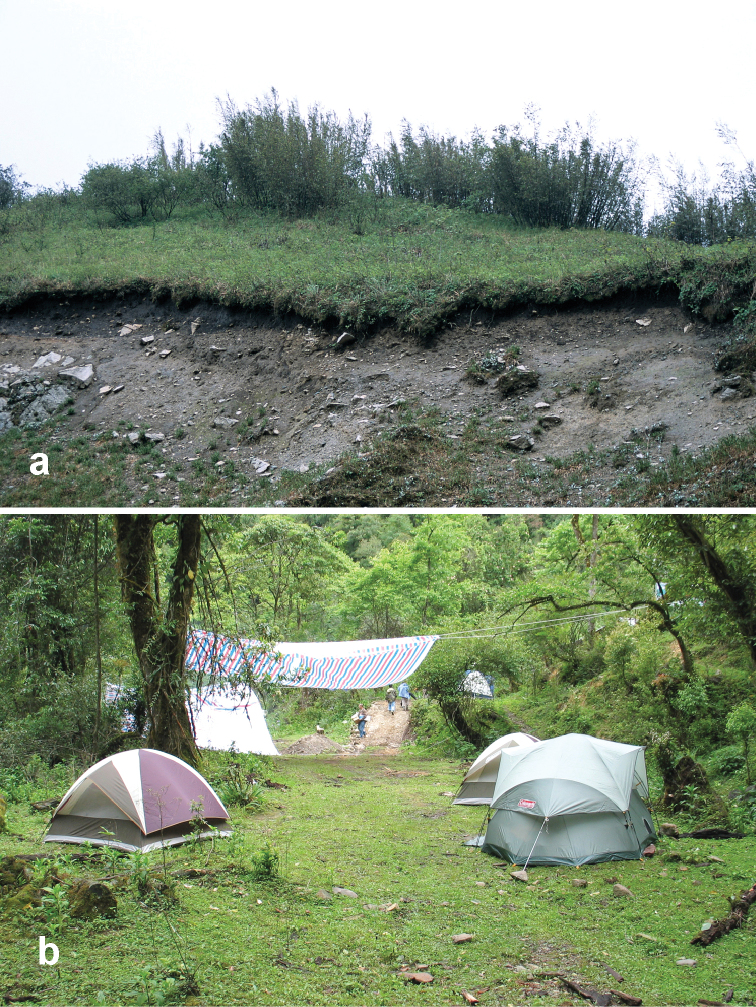
Photographs of habitats for *Amara* species in the Gaoligong Shan region. **a** Lushui County, Pianma Road just E of Fengxue Yakou, elevation 3150m; habitat in which specimens of *Amara birmana* and *Amara chalciope* were collected **b** Tengchong County, Longtang He at Dahetou Lingganjiao, elevation 2010 m; habitat in which specimens of *Amara birmana*, *Amara pingshiangi* and *Amara shaanxiensis* were collected.

**Figure 26.**
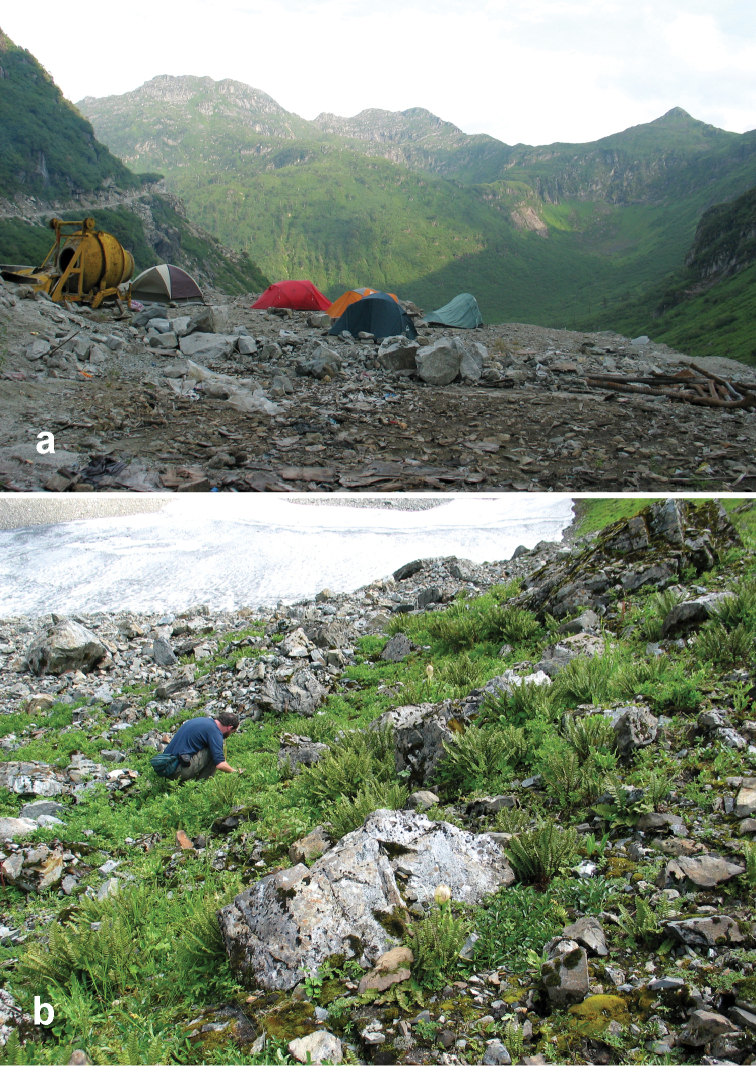
Photographs of habitats for *Amara* species in the Gaoligong Shan region. **a** Gongshan County, Heipu Yakou just E of Tunnel, elevation 3365 m; habitat in which specimens of *Amara chalciope* and *Amara elegantula* were collected **b** Gongshan County, southwest slope of Kawakarpu Shan on slope abiove and NE of Chukuai Lake, elevation 3950 m; habitat in which specimens of *Amara elegantula* were collected.

**Figure 27.**
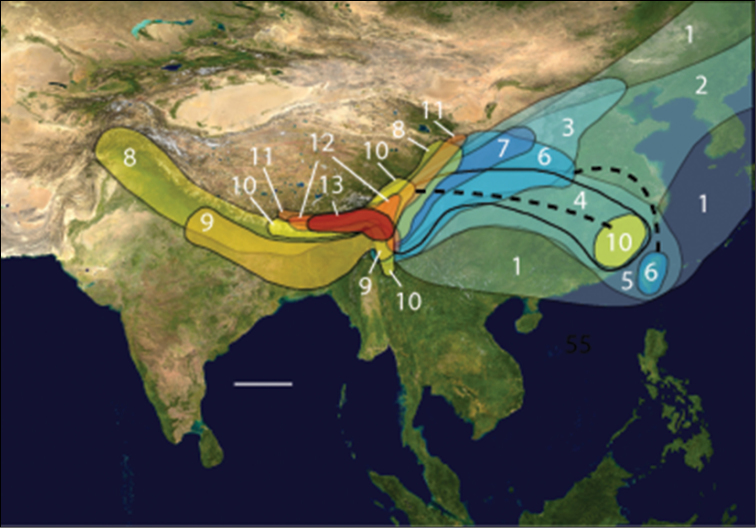
Map showing approximate overall geographical distributions of species occurring in the Gaoligong Shan region (ranges that extend to the upper right corner of the map are truncated there but continue northeast to Japan and the Russian Far East); dashed lines connect apparently significant disjunct areas of the range of a species; scale line = 500 km. **1** *Amara (Amara) congrua* Morawitz **2** *Amara (Bradytus) simplicidens* Morawitz **3** *Amara (Zezea) davidi* Tschitschérine **4** *Amara (Bradytus) pingshiangi* Jedlička **5** *Amara (Reductocelia) lucidissima* Baliani **6** *Amara (Amara) silvestrii* Baliani **7** *Amara (Amara) shaanxiensis* Hieke; **8** *Amara (Xenocelia) sikkimensis* Andrewes **9** *Amara (Harpaloamara) latithorax* Baliani **10** *Amara (Bradytus) chalciope* (Bates) **11** *Amara (Bradytus) dissimilis* Tschitschérine **12** *Amara (Pseudoamara) birmana* Baliani; **13** *Amara (Bradytus) elegantula* Tschitschérine.

**Figure 28. F26:**
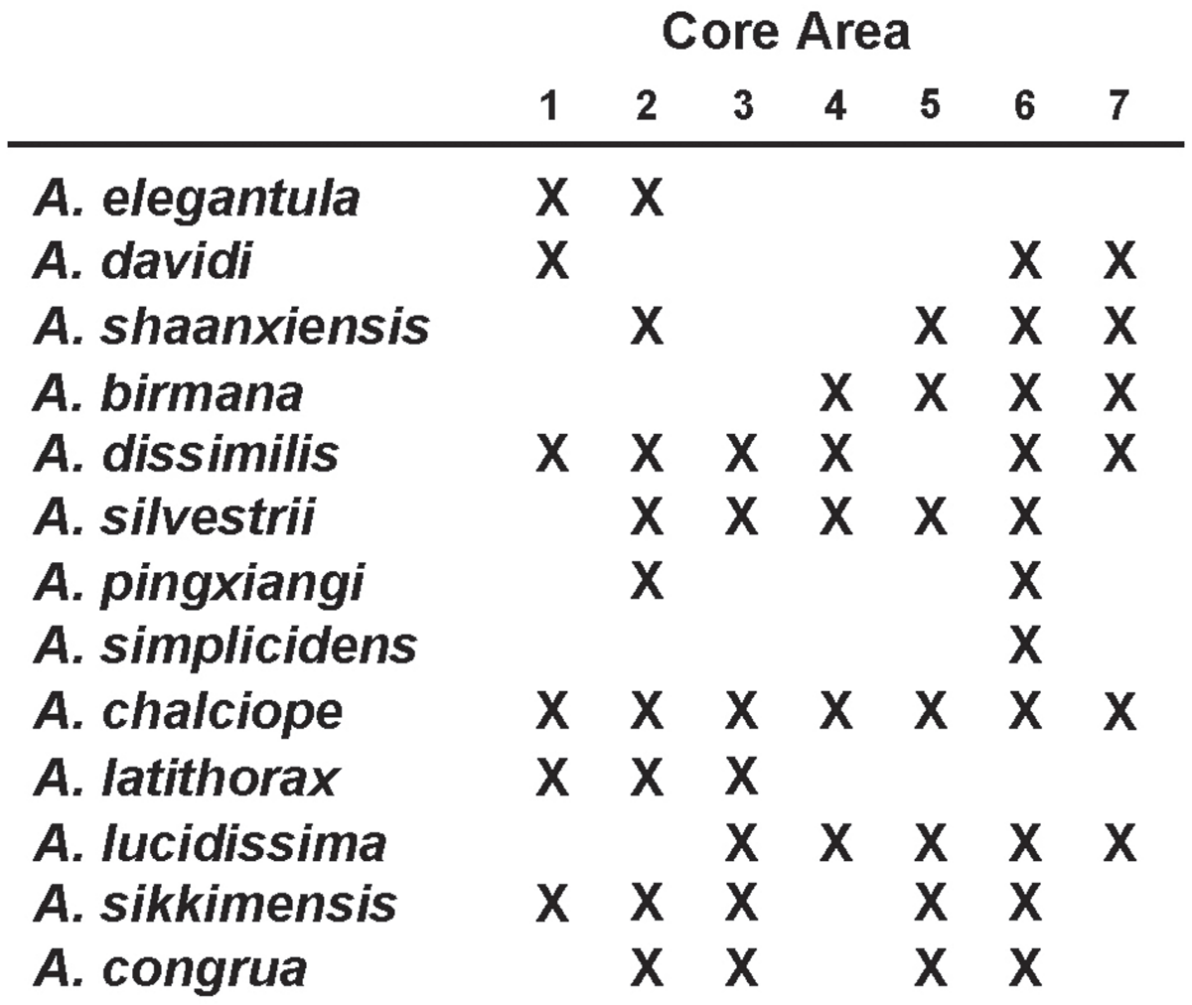
Chart showing the representation of *Amara* species in project-designated Core Areas (see Fig. 3) in the Gaoligong Shan region.

**Figure 29. F27:**
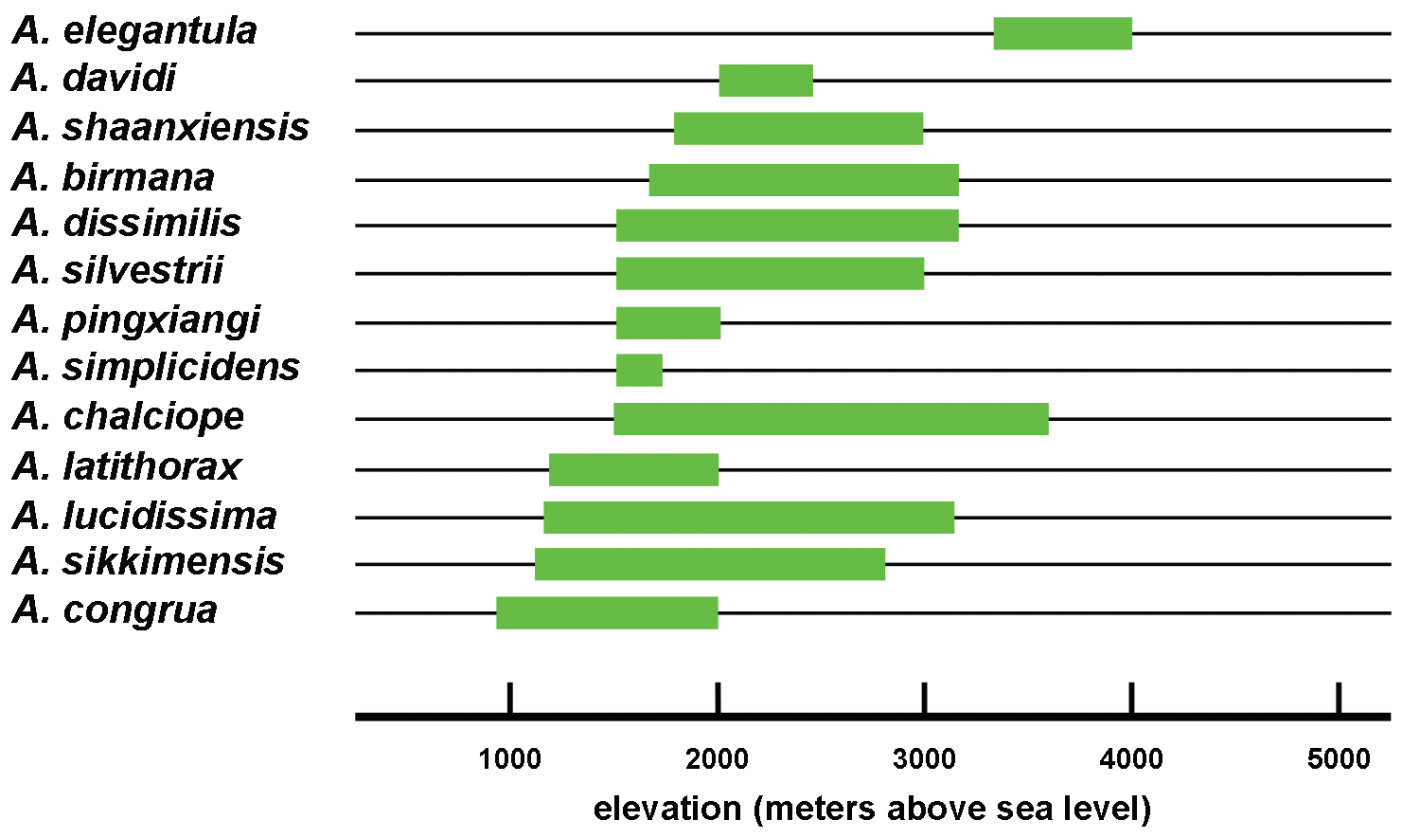
Chart illustrating the altitudinal ranges of *Amara* species represented in the Gaoligong Shan region.

**Figure 30.**
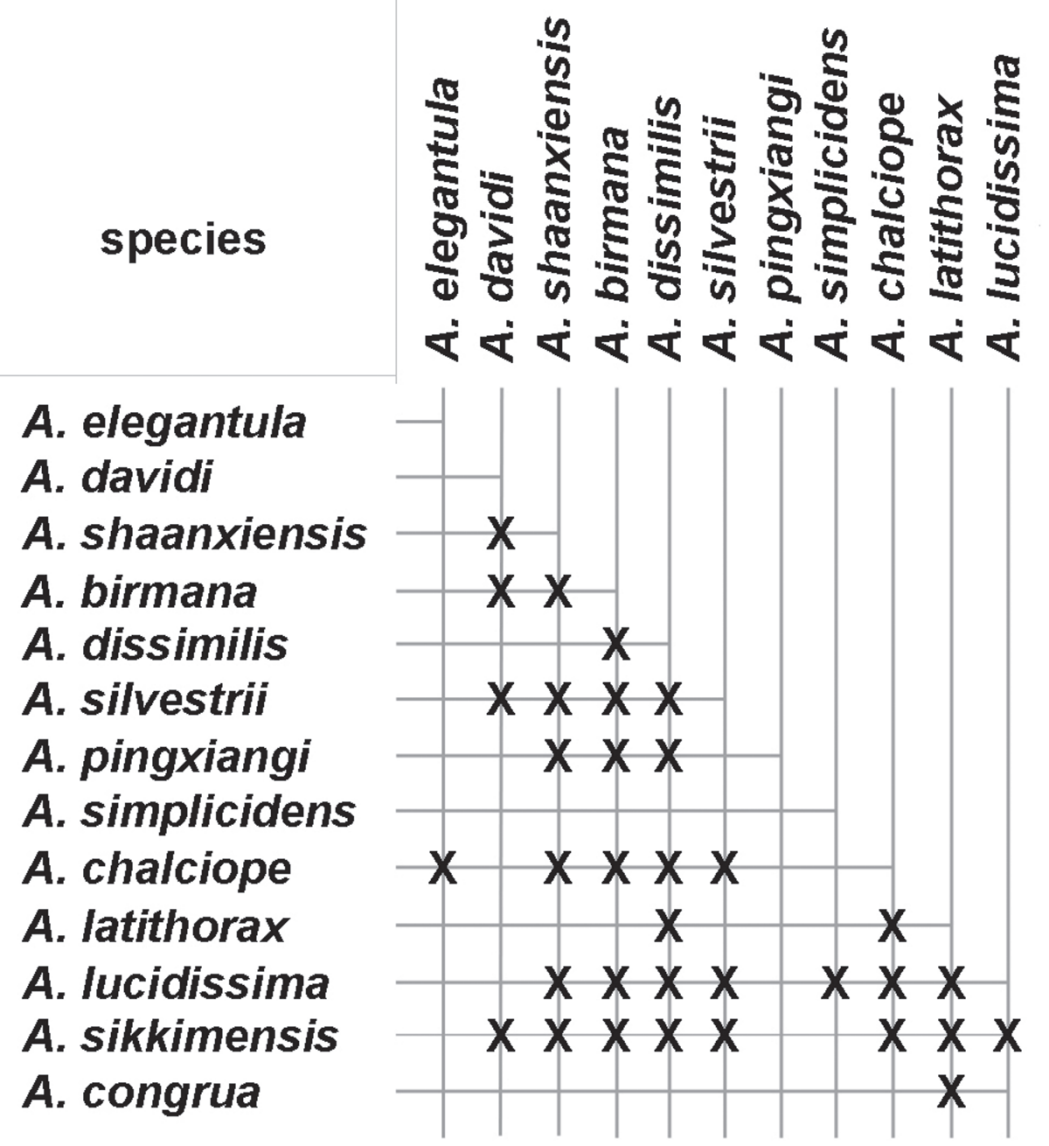
Chart illustrating the co-occurence (syntopy) of *Amara* species in samples from the same habitats and at the same sites in the Gaoligong Shan region.

## Discussion

Although the Gaoligong Shan region is at the heart of one of the world’s biodiversity hotspots (Myers et al. 2000), this area is near the southern limit of the geographical range of genus *Amara* in Asia; and so it is not surprising that species diversity there is less than in comparable areas farther north. Of the 13 species we recorded from the area based on material from our ten years of sampling plus additional records from collections (see list in Materials and Methods section above), none of these species are new to science or endemic to the area. Nonetheless, the composition of the *Amara* fauna of the area is of interest on several levels.

**Broad geographical distribution patterns.** The overall geographical ranges of the 13 species, superimposed on one another, are graphically approximated in Fig. 27. Among the geographical ranges of these species, three general range patterns are apparent. The first pattern includes seven species (*Amara congrua*, *Amara davidi*, *Amara lucidissima*, *Amara pingshiangi*, *Amara shaanxiensis*, *Amara sikkimensis* and *Amara simplicidens*), more or less broadly distributed in China and eastern Asia, but all of which have their western range limits at or near the Gaoligong Shan region. Two of these, *Amara congrua* and *Amara simplicidens*, have ranges that extend east and north to Japan and the Russian Far East, as well as across most of China east of the study area. Strangely, members of these two species were among the least frequently encountered *Amara* in the area during our study. The ranges of four species with eastern distributional ranges, *Amara congrua*, *Amara simplicidens*, *Amara pingshiangi* and *Amara lucidissima*, include Fujian Province in China. There are gaps in the known distributions of all of these species between the study area and Fujian, and inadequate sampling is likely the cause of these apparent gaps. For three species, *Amara congrua*, *Amara lucidissima* and *Amara silvestrii*, known geographical ranges also include Taiwan. Both *Amara congrua* and *Amara lucidissima* are also known from Fujian, right across the South China Sea from Taiwan, a disjunction of only about 250 to 400 km, depending on localities compared. The nearest Taiwanese localities for *Amara silvestrii* are more than 1400 km distant from the nearest mainland sites, in Shaanxi Province, and this may represent a real and significant range disjunction. The remaining species included among the class of eastern patterns, *Amara shaanxiensis*, has the most restricted known geographical distribution, recorded only from Yunnan and from Shaanxi Province, but it likely also occurs in intervening areas in along the southern edge of the Qinghai-Xizang Plateau in Sichuan and Gansu Provinces.

The second general geographical range pattern includes two species, *Amara elegantula* and *Amara latithorax*, with geographical ranges that include only the Himalayan region immediately to the west and the Gaoligong Shan region, where they reach their eastern distributional limit. *Amara elegantula* ranges as far west as eastern Nepal and adjacent parts of Xizang Autonomous Region (Tibet), and the range of *Amara latithorax* also extends west to Nepal, but apparently only on the southern slope of the Himalaya (i.e., it has not been recorded from the northern side of that range). The third general range pattern includes the remaining four species, *Amara birmana*, *Amara chalciope*, *Amara dissimilis* and *Amara sikkimensis*. The geographical distributions of each of these species include the Gaoligong Shan region and areas of varied distance to both the northwest and the northeast along the Himalayan range and the southern edge of the Qinghai-Xizang Plateau, respectively. Of these, *Amara sikkimensis* has the broadest distribution, extended from northen Pakistan in the west to Gansu Province in the east. The known range of *Amara chalciope* extends from Bhutan and Xizang Autonomous Region in the west to Sichuan in the east, with a significantly disjunct occurrence also in Fujian Province in southeastern China. Populations currently included under this name actually may represent a complex of species, a solid understanding of which will require additional study. The known range of *Amara dissimilis* extends from Xizhang in the northwest to Shaanxi in the northeast and actually includes parts of the Qinghai-Xizang Plateau itself in southern Qinghai Province. Finally, *Amara birmana* has the most restricted range of the species with this pattern, known only from northeastern India and northern Myanmar in the west to Sichuan in the east.

On first glance, the observed diversity of *Amara* species in the study area appears to be due simply to an overlap of faunal elements from both the east and the west. Certainly several of the species with broad distributions in large parts of eastern Asia (e.g., *Amara congrua* and *Amara simplicidens*) likely have their origins in regions to the northeast and so their occurrence in the study area is likely the results of range expansion into the region. However, even a casual look at the majority of range patterns illustrated in Fig. 27 tempts us to suggest that the Gaoligong Shan region, as a core part of the Hengduan Mountains System, may have served as an area of origin for at least part of the *Amara* fauna which now also occupies the Himalayan Ranges and southern edge of the Qinghai-Xizang Plateau to the west and east, respectively. The Hengduan Mountains date their origins to the late Mesozoic, whereas the uplift of the Himalayan Ranges and Qinghai-Xizang Plateau began later, in the early Cenozoic (Chaplin 2006). So this region may have been an area of differentiation, speciation and origin of montane elements from which, rather than to which, at least some of the species now ranging more broadly in the region subsequently spread. However, an understanding of phylogenetic relationships among the Eurasian and Oriental *Amara* species is required in order to test this hypothesis, and such an analysis has not yet been undertaken. Comparative DNA sequence data in particular should be highly informative for resolving these relationships. Schmidt et al. (2012) provided distributional and molecular phylogenetic evidence that together support a “Tibetan origin” for the Himalayan endemic *Ethira* clade of Pterostichini, rather than an origin by dispersal either from western Asia or from mountainous areas to the east of the Himalaya, as we suggest here for *Amara*. However, because they could not determine the sister group of the *Ethira* clade from their analysis, it remains unclear whether more inclusive (i.e., older, more basal) relationships are with some element of the pterostichine fauna of the temperate north (as they suggest) or not. Pterostichine material from the Gaoligong Shan inventory project is currently under study, and it will be interesting to see if this material provides any new insights into the origins of that element of the present faunas of the Himalayan Ranges and southern edge of the Qinghai-Xizang Plateau.

**Regional geographical and altitudinal distribution patterns.** Within the Gaoligong Shan study area, most of the species represented are broadly distributed, both geographically and altitudinally. This is not surprising given the shared preferences of their members for open and disturbed habitats. Such areas are abundant in the lowlands, most often associated with agriculture and human habitation. They are also abundant at all elevations within in the Gaoligong Shan itself, whether in naturally open alpine areas above tree line or in open areas along streams, on flood outwash flats or stabilized landslides, or in open sites created by humans, such as along road cuts, in forest clearcuts and previously burned areas and around all forms of human settlement and activity. What is perhaps unexpected is that a few of the species are quite restricted in their geographical and altitudinal ranges within the study area.

The chart in Fig. 28 summarizes the recorded regional distributions of the species with respect to our project-designated Core Areas; and the recorded altitudinal ranges for each species are shown in Fig. 29. Only *Amara chalciope* is recorded from all seven Core Areas, and this species also shows the broadest altitudinal range (from 1500 to just over 3600 m). *Amara dissimilis* is recorded from six of the Core Areas (not yet recorded from Core Area 5) and also has a broad altitudinal range (from 1515 to 3150 m). Two species, *Amara silvestrii* and *Amara sikkimensis*, are recorded from five of the Core Areas, including both the northernmost and southernmost, so they are likely to be found in the other Core Areas with additional sampling. Both species also occupy broad altitudinal ranges (from 1515 to 3000 m and from 1175 to 2800 m, respectively). Four species have been recorded from fewer Core Areas but nonetheless are likely to be found more widely in the region with additional sampling in gap areas in their ranges. *Amara davidi* has been recorded from three Core Areas (1, 6 and 7), *Amara shaanxiensis* from four (2, 5, 6 and 7), *Amara pingshiangi* from two (2 and 6) and *Amara congrua* from four (2, 3, 5 and 6). Among these, *Amara shaanxiensis* has the broadest recorded altitudinal range (from just over 1800 to 3000 m) and *Amara davidi* and *Amara pingshiangi* the narrowest (from 2020 to 2440 m and from 1515 to 2010 m, respectively). *Amara congrua* has a moderately broad recorded altitudinal range (from 892 to 2000 m), including the lowest elevation at which any *Amara* species has been recorded in the study area. The five species not yet mentioned all have geographical and, in some cases, also altitudinal ranges that are restricted to at least some extent. Among these, *Amara lucidissima* is the most widespread, both geographically (Core Areas 3–7) and altitudinally (from 1185 to 3140 m); and because it has been locally abundant wherever it has been found, the absence of records from the northern Core Areas (1 and 2) is significant. Similarly, *Amara birmana* has been recorded only from the southern half of the study area (Core Areas 4-7), at elevations ranging from 1590 to 3150 m, and this is likely a true measure of its actual distribution in the region. Two species, *Amara elegantula* and *Amara latithorax*, have been recorded only from the northern part of the study area (in Core Areas 1 and 2 and 1-3, respectively). However, these two differ markedly in their altitudinal ranges, with *Amara latithorax* recorded at elevations ranging from about 1200 to 2000 m and *Amara elegantula* found only at elevations ranging from 3350 to at least 4000 m. For *Amara elegantula*, a geographical range restricted to the north is expected, given its restricted high-elevation altitudinal range and the physiography of the study area, with virtually all contiguous areas above 3300 m found in the north. However, we find the restricted northern distribution of *Amara latithorax* surpising, given that its altitudinal range requirements seemingly could be met virtually anywhere along the length of the Gaoligong Shan. The remaining species, *Amara simplicidens*, has been recorded only from Core Area 6 in the southwestern part of the study area and only at relatively low elevations (from 1515 to 1740 m). This is somewhat surprising given that the overall geographical range of this species is so large, extended eastward all the way to Japan and the Russian Far East.

A comparison of recorded diversity for *Amara* species among the seven Core Areas (Fig. 28) shows that all areas are occupied by at least five species, with highest diversity in Core Area 6 (with 11 of the 13 species), second highest in Core Area 2 (with 9 species) and lowest recorded diversity in Core Area 4 (which is also by far the smallest Core Area). Core Areas 1 and 2 uniquely share one species, *Amara elegantula*, and Core Areas 1-3 together are uniquely occupied two species (*Amara elegantula* and *Amara latithorax*). Core areas 3-7 together are uniquely occupied by three species, *Amara birmana*, *Amara lucidissima* and *Amara simplicidens*; and Core Areas 4-7 together uniquely include two of these, *Amara birmana* and *Amara simplicidens*. Only Core Area 6 has a species, namely *Amara simplicidens*, not shared with any other Core Area. Consequently, but perhaps not surprisingly, the northern and southern extremes of the study area (Core Areas 1 and 2 in the north and 4-7 in the south) have the most distinctive *Amara* faunas, with Core Area 3 apparently representing a region of faunal transition.

With respect to the altitudinal distribution of the *Amara* fauna of the study area (Fig. 29), several points can be made. Highest species diversity is concentrated at about 2000 m in elevation, with 11 of the 13 species recorded from that elevation. Only *Amara elegantula* and *Amara simplicidens* do not occur at that elevation, the former only far above that level, the latter only below it. Three species, *Amara congrua*, *Amara latithorax* and *Amara pingshiangi* reach their upper altitudinal limit at about this level, and one, *Amara davidi*, reaches its lower limit. The altitudinal ranges of only four species (*Amara congrua*, *Amara latithorax*, *Amara lucidissima* and *Amara sikkimensis*) extend below about 1200 m, and those of only two species (*Amara chalciope* and *Amara elegantula*) include areas above 3200 m. It could be quite informative to monitor future changes in the altitudinal ranges of these species with respect to the baseline data recorded here as one measure of climate change.

**Syntopy of species in the regional fauna.** Because virtually all *Amara* species prefer habitats that are open and more or less disturbed, either by natural processes or through human activities, and because there is so much overlap in both the geographical and altitudinal ranges of species represented in the study area, it is not at all surprising to find that specimens of several different species can be found together at the same site in the same habitat (i.e., syntopic). This co-occurrence of species at sites in the region is summarized in Fig. 30. During fieldwork for this project, it was not uncommon to find several species, up to seven in at least one instance, together at the same time at the site. Four species, *Amara birmana*, *Amara chalciope*, *Amara lucidissima* and *Amara sikkimensis*, have been recorded syntopic with eight other species in one or more sites; three species, *Amara dissimilis*, *Amara shaanxiensis* and *Amara silvestrii*, have been found syntopic with seven other species; and *Amara davidi*, *Amara latithorax* and *Amara pingshiangi* have been recorded syntopic with four, five and three other species each, respectively. Members of these ten species are often found together, in various combinations, at different sites in the study area, although not one of them co-occurs with all the others. By contrast, three species have been found syntopic with just one other species. *Amara congrua* adults have been found together with those of *Amara latithorax* one time (in Shanga, Fugong County). Members of *Amara simplicidens* have been found just once with those of *Amara lucidissima* (near Datang Village, Tengchong County). Finally, adults of *Amara elegantula* have been found twice with those of *Amara chalciope* twice, both at sites around Heipu Yakou (Pass) on the Gongshan-Dulong Road at an elevation of 3365 m, where the altitudinal ranges of these species overlap narrowly. It is possible that *Amara congrua* and *Amara simplicidens* will be found co-occurring more broadly with the same species or with other species in the future because the geographical and/or altitudinal ranges of each are within the ranges of other species, whereas the restricted geographical and altitudinal ranges of *Amara elegantula* should preclude any but the already observed co-occurrence with *Amara chalciope*.

In the future, it will be interesting to compare and contrast both the broad and regional geographical distributions and the altitudinal and ecological range patterns seen in other carabid groups represented in the area with those found among the *Amara* species and reported here.

## Supplementary Material

XML Treatment for Amara (Zezea) davidi

XML Treatment for Amara (Amara) congrua

XML Treatment for Amara (Amara) silvestrii

XML Treatment for Amara (Amara) shaanxiensis

XML Treatment for Amara (Pseudoamara) birmana

XML Treatment for Amara (Xenocelia) sikkimensis

XML Treatment for Amara (Harpaloamara) latithorax

XML Treatment for Amara (Bradytus) chalciope

XML Treatment for Amara (Bradytus) dissimilis

XML Treatment for Amara (Bradytus) elegantula

XML Treatment for Amara (Bradytus) simplicidens

XML Treatment for Amara (Bradytus) pingshiangi

XML Treatment for Amara (Reductocelia) lucidissima
